# Microbiota Transfer Therapy alters gut ecosystem and improves gastrointestinal and autism symptoms: an open-label study

**DOI:** 10.1186/s40168-016-0225-7

**Published:** 2017-01-23

**Authors:** Dae-Wook Kang, James B. Adams, Ann C. Gregory, Thomas Borody, Lauren Chittick, Alessio Fasano, Alexander Khoruts, Elizabeth Geis, Juan Maldonado, Sharon McDonough-Means, Elena L. Pollard, Simon Roux, Michael J. Sadowsky, Karen Schwarzberg Lipson, Matthew B. Sullivan, J. Gregory Caporaso, Rosa Krajmalnik-Brown

**Affiliations:** 1 Biodesign Swette Center for Environmental Biotechnology, Arizona State University, Tempe, AZ 85287 USA; 2 School for Engineering of Matter, Transport and Energy, Arizona State University, Tempe, AZ 85287 USA; 3 Soil, Water and Environmental Sciences, University of Arizona, Tucson, AZ 85721 USA; 4 Centre for Digestive Diseases, Five Dock, NSW 2046 Australia; 5 Department of Ecology and Evolutionary Biology, University of Arizona, Tucson, AZ 85287 USA; 6 Mucosal Immunology and Biology Research Center, Massachusetts General Hospital for Children, Boston, MA 02114 USA; 7 Division of Gastroenterology, Department of Medicine, University of Minnesota, Minneapolis, MN 55455 USA; 8 BioTechnology Institute, University of Minnesota, St. Paul, MN 55108 USA; 9 Center for Immunology, University of Minnesota, Minneapolis, MN 55414 USA; 10 Integrative Developmental Pediatrics, Tucson, AZ 85701 USA; 11 Department of Soil, Water and Climate, University of Minnesota, St. Paul, MN 55108 USA; 12 Pathogen and Microbiome Institute, Northern Arizona University, Flagstaff, AZ 86011 USA; 13 Department of Biological Sciences, Northern Arizona University, Flagstaff, AZ 86011 USA; 14 School of Sustainable Engineering and the Built Environment, Arizona State University, Tempe, AZ 85287 USA; 15 Department of Microbiology, Ohio State University, Columbus, OH 43210 USA; 16 Department of Civil, Environmental and Geodetic Engineering, Ohio State University, Columbus, OH 43120 USA

**Keywords:** Autism spectrum disorders (ASD), Fecal microbiota transplant (FMT), Clinical trial, Gut bacteria, Gut bacteriophage, Microbiome, Virome

## Abstract

**Background:**

Autism spectrum disorders (ASD) are complex neurobiological disorders that impair social interactions and communication and lead to restricted, repetitive, and stereotyped patterns of behavior, interests, and activities. The causes of these disorders remain poorly understood, but gut microbiota, the 10^13^ bacteria in the human intestines, have been implicated because children with ASD often suffer gastrointestinal (GI) problems that correlate with ASD severity. Several previous studies have reported abnormal gut bacteria in children with ASD. The gut microbiome-ASD connection has been tested in a mouse model of ASD, where the microbiome was mechanistically linked to abnormal metabolites and behavior. Similarly, a study of children with ASD found that oral non-absorbable antibiotic treatment improved GI and ASD symptoms, albeit temporarily. Here, a small open-label clinical trial evaluated the impact of Microbiota Transfer Therapy (MTT) on gut microbiota composition and GI and ASD symptoms of 18 ASD-diagnosed children.

**Results:**

MTT involved a 2-week antibiotic treatment, a bowel cleanse, and then an extended fecal microbiota transplant (FMT) using a high initial dose followed by daily and lower maintenance doses for 7–8 weeks. The Gastrointestinal Symptom Rating Scale revealed an approximately 80% reduction of GI symptoms at the end of treatment, including significant improvements in symptoms of constipation, diarrhea, indigestion, and abdominal pain. Improvements persisted 8 weeks after treatment. Similarly, clinical assessments showed that behavioral ASD symptoms improved significantly and remained improved 8 weeks after treatment ended. Bacterial and phagedeep sequencing analyses revealed successful partial engraftment of donor microbiota and beneficial changes in the gut environment. Specifically, overall bacterial diversity and the abundance of *Bifidobacterium*, *Prevotella*, and *Desulfovibrio* among other taxa increased following MTT, and these changes persisted after treatment stopped (followed for 8 weeks).

**Conclusions:**

This exploratory, extended-duration treatment protocol thus appears to be a promising approach to alter the gut microbiome and virome and improve GI and behavioral symptoms of ASD. Improvements in GI symptoms, ASD symptoms, and the microbiome all persisted for at least 8 weeks after treatment ended, suggesting a long-term impact.

**Trial registration:**

This trial was registered on the ClinicalTrials.gov, with the registration number NCT02504554

**Electronic supplementary material:**

The online version of this article (doi:10.1186/s40168-016-0225-7) contains supplementary material, which is available to authorized users.

## Background

Autism spectrum disorders (ASDs) are complex neurobiological disorders that impair social interactions and communication and lead to restricted, repetitive, and stereotyped patterns of behavior, interests, and activities [1]. While ASD diagnoses are increasing, with ~1–2% of children currently diagnosed worldwide [2], the causes of this disorder remain poorly understood and appear to involve a complex interplay of genetic and environmental factors, of which the microbiome is an environmental factor that is partially inherited from the mother [3]. Despite increased ASD diagnoses, there remains no US Food and Drug Administration (FDA)-approved pharmaceutical treatment to alleviate core ASD symptoms [4]. Coincident with ASD, many children and adults also experience significant gastrointestinal (GI) symptoms, such as constipation, diarrhea, and alternating constipation/diarrhea [5], which correlate with ASD severity [6, 7]. Such GI symptoms appear to be due, in part, to dysbiotic gut microbiota [8] and perhaps their missing roles on modulating metabolites (e.g., 4-ethylphenylsulfate, indolepyruvate, and corticosterone) that affect GI function and neurobiological conditions, such as ASD and anxiety [9, 10]. Many children with ASD often undergo increased oral antibiotic treatment during the first 3 years of life [11], which is thought to destabilize their gut microbiota [12] and open opportunities for competitive potential pathogens to contribute to ASD severity [13, 14]. A number of studies reported that children with ASD have altered gut bacteria profiles compared with neurotypcial children [13–18], although in certain cohorts, no significant difference has been reported [19, 20]. Because children with ASD have lower abundances of fermentative bacteria (e.g., *Prevotella copri*), and lower overall bacterial diversity, it has also been hypothesized that lack of beneficial gut microbiota impairs neurological health [21]. Consistent with this, experiments done in an ASD mouse model demonstrated that augmentation with *Bacteroides fragilis* alone could alter gut microbiota and blood metabolite profiles, correct increased gut permeability (gaps in cell-to-cell junctions), and improve ASD-associated behaviors [9]. In children with ASD, a small open-label study found that 8 weeks of treatment with oral vancomycin (a non-absorbable antibiotic which acts only in the gut) led to major improvements in both GI symptoms and ASD symptoms, although the benefits were lost within a few weeks after treatment was stopped [22]. Thus, gut microbiota appears strongly associated with ASD. Viruses are also abundant in the gut [23] and may also impact ASD symptoms by modulating the abundance, evolutionary trajectories, and metabolic outputs of gut microbiota like they do in other environments [24].

Interest in rebalancing human gut microbiota to treat disease is growing [25]. Diet, antibiotics, probiotics, prebiotics, and fecal microbiota transplants are treatments with reported potential [26–30]. For ASD, however, only temporary symptom improvements have been reported from vancomycin treatment [22], and probiotics have had mixed clinical results with minimal microbiota analysis or long-term follow-up [31]. Contrasting to probiotics which contain a few bacterial species from milk cultures, fecal microbiota transplant (FMT) contains approximately a thousand bacterial species native to the gut and has helped treat recurrent *Clostridium difficile* infection [32] and is promising for the treatment of chronic inflammatory diseases such as inflammatory bowel disease [33] and insulin sensitivity [34]. Therefore, ASD’s GI and behavioral symptoms may derive, at least in part, from gut microbiota dysbiosis and FMT may effectively rebalance the gut microbiota and alleviate some GI and ASD symptoms.

FMT therapy usually involves only a single dose for recurrent *C. difficile* infection [32] and other GI conditions, although there is a growing interest in the use of several doses [35]. For this study, a prolonged, daily treatment regimen was implemented based on the clinical experiences of team member Thomas Borody who found that only *C. difficile* infection is responsive to one or two FMT infusions. All other GI problems—originally described in ulcerative colitis [36]—require multiple infusions of donor microbiota to achieve measurable and long-lasting benefits, including those associated with ASD. An open-label trial was designed to investigate the safety, tolerability, and efficacy of FMT for GI and behavior symptoms in children with ASD. Long-term FMT treatment was administered to 18 children with GI problems and ASD. Clinical responses, gut bacteria, and phage double-stranded DNA profiles were monitored for 18 weeks. Briefly, a modified FMT protocol, termed Microbiota Transfer Therapy (MTT), involved 14 days of oral vancomycin treatment followed by 12–24 h fasting with bowel cleansing, then repopulating gut microbiota by administering a high initial dose of Standardized Human Gut Microbiota (SHGM) [37] either orally or rectally followed by daily, lower maintenance oral doses with a stomach acid suppressant for 7–8 weeks. A stomach-acid suppressant was used to increase the survival of SHGM through the stomach. Participants were followed for an additional 8 weeks after treatment ended, to determine if treatment effects were temporary or long-lasting. This report focuses on the safety and tolerability of MTT and its effects on microbiota, GI symptoms, and other ASD-related symptoms.

## Methods

### Goal

The goals of the study were to follow gut microbiota in healthy and treated children with ASD longitudinally as well as to evaluate an investigational new treatment, MTT, for its effectiveness in children with ASD in treating both GI symptoms (primary outcome) and ASD-related symptoms (secondary outcomes), and to determine the effect of MTT on the gut microbiome.

### Study design

**Fig. 1:**
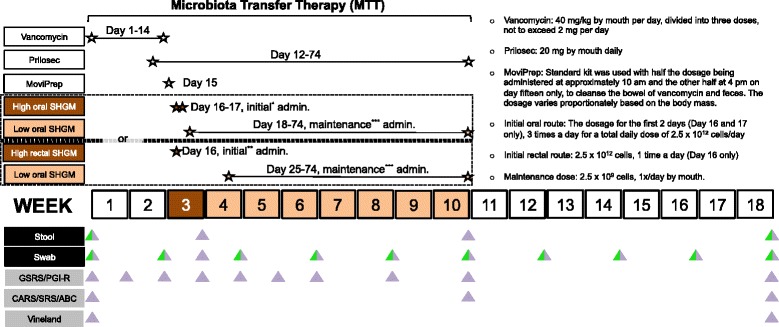
Study design timeline. The trial consists of 10-week Microbiota Transfer Therapy (MTT) and 8-week follow-up observation period after treatment stopped. Schematic timeline represents a series of treatments that were performed during MTT (*top*) and frequencies of sample collection and GI/behavior assessments (*bottom*; neurotypical and ASD group colored in *green* and *purple*, respectively)

The general study design was an open-label clinical trial involving 18 children with ASD (ages 7–16 years) who were diagnosed by the Autism Diagnostic Interview-Revised (ADI-R) and had moderate to severe gastrointestinal problems. FDA limited our pilot study to older children ages 7–17 years, since most FMT studies have been conducted on adults, and there was very limited data and knowledge of the impact and usage of FMT for younger children. Each child participated in the study for 18 weeks in total, consisting of a 10-week MTT treatment and an 8-week follow-up observation period after the treatment stopped. As a control group, 20 age- and gender-matched neurotypical children without GI disorders were recruited. Neurotypical children were monitored for 18 weeks but not treated. For FMT treatment, two routes of administration were compared, oral versus rectal, for the initial dose, followed by a lower maintenance dosage given orally for 7–8 weeks. Participants were randomly assigned to the two groups but allowed to switch if they had a strong preference or intolerance regarding the mode of administration. The researchers were not blinded to the group allocation or outcome assessment. Figure 1 illustrates the study design.

### Subject recruitment

The study physician first assessed inclusion-exclusion criteria through an extensive review of the participants’ last 2 years of medical records and height/weight/growth charts. Once qualified, autism spectrum diagnosis was verified using the ADI-R, which involved a phone interview of the parents by an ADI-R evaluator. Once qualified and enrolled, participants engaged in an initial 30-min meeting which included a general physical health examination by the study physician and discussion with a project staff member. Participant exclusion criteria included antibiotics use in the prior 6 months or probiotics use in the prior 3 months; dependence on tube feeding; severe GI problems that require immediate treatment (life-threatening); recent/scheduled surgeries; diagnosed as severely malnourished or underweight; and diagnosed with a single-gene disorder, major brain malformations, ulcerative colitis, Crohn’s disease, celiac disease, or eosinophilic esophagitis. None of the neurotypical children had been diagnosed with mental disorders including ASD, attention-deficit hyperactivity disorder (ADHD), depression, or anxiety. None of the neurotypical children had first-degree relatives (i.e., parents and siblings) with ASD. From participants, initial blood and stool samples were collected. Parents were asked to complete a 1-week diet assessment on behalf of their child at the beginning of the study. Participants were recruited primarily from the greater Phoenix, Arizona area; three were from outside that area. Neurotypical families were recruited from friends of the ASD families and professionals who work with ASD families.

### Intervention

The MTT treatment protocol consisted of four key parts: (1) oral vancomycin, (2) MoviPrep, (3) SHGM, and (4) Prilosec. As summarized in Fig. 1, the treatment began with 14 days of oral vancomycin, a non-absorbable broad spectrum antibiotic that stays in the GI tract. A 14-day course of vancomycin was used to ensure that pathogenic bacteria were profoundly suppressed. Prilosec (an acid pump inhibitor) was administered starting on the 12th day of vancomycin, and continued until the end of the lower dosage of SHGM in order to reduce stomach acidity and increase the survival rate of SHGM through the stomach. On day 15, parents administered MoviPrep, a drink that flushes the bowels, to remove most remaining gut bacteria and vancomycin. To enhance its effectiveness, a fasting period of 1 day was implemented during which participants were only allowed to consume clear liquids (children under 12 years were allowed a light breakfast), and then at 4 pm and 8 pm, parents administered the two doses of MoviPrep. On day 16, the participants began either oral administration of SHGM (2.5 × 10^12^ cells/day) mixed in a chocolate milk, milk substitute, or juice for 2 days (divided into three daily doses), or a single rectal dose of SHGM (2.5 × 10^12^ cells), given similar to an enema. The rectal dose was administered slowly over 1 h, and participants remained prone for at least several hours, and delayed defecation for at least several hours. The rectal dose was administered under the direct supervision of the study physician, and the first oral dose was similarly administered in the presence of the physician. Participants were randomly assigned to either the oral or rectal route of administration. If one administration route was not tolerated, or if the family preferred the other route, then participants had the option of trying the other route. For participants who received the major initial rectal dose, they waited for 1 week (so the effect of the rectal dose could be evaluated by itself) and then received a lower oral maintenance dose (2.5 × 10^9^ cells) for 7 weeks. In contrast, for participants who received major initial oral doses, they received a lower oral maintenance dose (2.5 × 10^9^ cells) for 8 weeks, directly after the major initial oral dose. The lower maintenance SHGM doses were self-administered orally every day up to the end of week 10. After treatment was stopped, participants were monitored for another 8 weeks.

### Standardized human gut microbiota

Instead of pure stool, this study involved the use of standardized human gut microbiota that is > 99% bacteria and prepared as previously described using stool from healthy individuals as starting material [37]. Briefly, donors underwent rigorous screening that involved regular questionnaires, review of medical history, and physical examinations to rule out infectious disease, metabolic syndrome, gastrointestinal disorders, and neurologic or neurodevelopmental problems. Serologic testing was performed to rule out infection with human immunodeficiency virus-1 and -2; hepatitis A, B, and C; and syphilis. The stool used in preparation was tested for potential bacterial pathogens (*C. difficile* toxin B, *Campylobacter*, *Salmonella*, toxin-producing *Escherichia coli*, *Vibrio*, *Yersinia*, *Listeria*, methicillin-resistant *Staphylococcus aureus*, and vancomycin-resistant *Enterococcus*), potential parasites (Giardia, Cryptosporidium, Cyclospora, and Isospora), and potential viral infections (Rotavirus A, Adenovirus, and Norovirus). Metabolic health of donor individuals was assessed with physical examinations and serologic testing (fasting glucose, lipid panel, liver function tests, and high sensitivity C-reactive protein). In addition, the fluorescent antinuclear antibody was employed as a screen for autoimmunity risk. Any single abnormality resulted in disqualification of the donor and prevents material release. The donated material was then extensively filtered and standardized under anaerobic conditions, following FDA good manufacturing processes (GMP), resulting in > 99% microbiota. The final product was in liquid form which can be frozen and was proven to be highly effective for treating *C. difficile* [37]. The SHGM was stored in −80 °C freezers at Arizona State University (ASU), and then delivered to families on dry ice every week during the study. Families were instructed to keep the SHGM in a container with dry ice and thaw it shortly before use.

Participants received two different doses of SHGM; the high major dose and a lower maintenance dose. The high-dose SHGM was at a daily dosage of 2.5 × 10^12^ cells, with 2 days for oral and 1 day for rectal administration. The rationale for the high dose was that after the MoviPrep, 1-day fast is presumably the most critical time in which to provide new beneficial bacteria. The maintenance dose of SHGM for the following 7–8 weeks was 2.5 × 10^9^ cells/day.

### Evaluation and sample collection

Parents were asked to collect stool samples from their child on approximately 0, 21, 70, and 126 days and to collect fecal swabs bi-weekly on 0, 14, 21, 28, 42, 56, 70, 84, 98, 112, and 126 days. The stool samples were analyzed to determine the types and amounts of gut microbiota present. For safety tests, blood samples were collected on approximately 0, 19, 33, and 74 days. During the study, the participants met with the physician for an initial physical evaluation (including review of medical history) and following evaluations on 16, 30, and 74 days. The physician had a phone consult with families on 7, 21, 42, and 130 days, and more frequently if adverse symptoms occurred, or if families had any questions. Neurotypical participants did not receive any treatment. They simply provided stool samples (at weeks 0 and 19) and swab samples every 2 weeks for 4 months.

### Assessments of gastrointestinal symptoms

Parents/guardians were asked to fill in the Gastrointestinal Symptom Rating Scale (GSRS) and the daily stool records (DSR). The GSRS is an assessment of GI symptoms during the previous week, based on 15 questions, which are then scored in five domains: abdominal pain, reflux, indigestion, diarrhea, and constipation. A score for each domain was reported based on the average within the questions in that domain. The original GSRS used a 4-point scale, but this study employed a revised version which included 7-point Likert scale which also has simpler language [38]. The GSRS were assessed on 0, 7, 14, 21, 28, 35, 42, 56, 74, and 130 days, and the children with ASD were defined as non-responders when they achieved less than 50% reduction in the average GSRS. The baseline DSR was collected daily, for 2 weeks, during the treatment phase, and the last 2 weeks of the observation period. The DSR primarily included a rating of the stool using the Bristol Stool Form scale (1 = very hard, 7 = liquid).

### Assessments of autism and related symptoms

The ADI-R is a 2-h structured interview and is one of the primary tools used for clinical diagnosis of autism and autism spectrum disorders. It is not designed to be a measure of autism severity but higher scores are generally consistent with more severe symptoms [39]. The ADI-R was used to verify the diagnosis of ASD for admission into the study. The Parent Global Impressions-III (PGI-III) was introduced here as an expanded version of PGI-R [40] by using a 7-point scale ranging from “much worse” to “much better.” An “average change” is calculated by computing the average in all 18 scores of the PGI-III-final. This tool was chosen because it was found to be more reliable to ask parents directly about observed changes than to have them estimate symptom severity at beginning and end and then compute a difference [40]. Also, the use of a 7-point scale to detect changes seems to yield a high sensitivity to changes. The Childhood Autism Rating Scale (CARS) is a 15-item scale that can be used to both diagnose autism and ASD and assess the overall severity of the symptoms. The Aberrant Behavior Checklist (ABC) assesses problem behaviors in five areas common in children with ASD, including irritability, lethargy, stereotypy, hyperactivity, and inappropriate speech. The Social Responsiveness Scale (SRS) is a 65-item scale that assesses social impairments, a core issue in autism, including social awareness, social information processing, capacity for reciprocal social communication, social anxiety/avoidance, and autistic preoccupations and traits. The Vineland Adaptive Behavior Scale II (VABS-II) is a measure of the functioning level in four different domains: communication, daily living skills, socialization, and motor skills, and 11 sub-domains. The raw scores were converted into an age equivalent score. Its assessment of adaptive skills complements the ABC, which assesses problem behaviors.

PGI-III on 0, 7, 14, 21, 28, 35, 42, 56, 74, and 130 days and the CARS, ABC, and SRS at baseline, at the end of treatment, and at the end of the observation period were assessed, whereas the VABS-II was assessed at baseline and at the end of the observation period only, because it is lengthy and likely less sensitive to short time periods since it assesses changes in specific adaptive skills. The same professional evaluator assessed the ADI-R and the CARS, and parents assessed the PGI-III, ABC, SRS, and VABS-II.

### Microbial DNA extraction and next-generation sequencing

Microbial DNA was extracted from feces, swabs, and donor samples using the PowerSoil® DNA Isolation Kit (Mobio Carlsbard, CA). A 16S rRNA library for MiSeq Illumina platform was prepared according to the protocol from Earth Microbiome Project (http://www.earthmicrobiome.org/emp-standard-protocols/). The barcoded primer set 515f-806r were used for pair-ended sequencing to target the 16S rRNA V4 region [41]. Library preparation and sequencing work were performed at the Microbiome Analysis Laboratory in the Swette Center for Environmental Biotechnology (http://krajmalnik.environmentalbiotechnology.org/microbiome-lab.html). These primers amplify both bacterial and archaeal 16S rRNA genes. Archaea-specific changes were not observed and are not discussed in this manuscript.

### Microbiome bioinformatics

Microbiome sequencing data were analyzed using Quantitative Insights Into Microbial Ecology (QIIME) 1.9.1 [42], biom-format version 2.1.5 [43], VSEARCH version 1.7.0 (https://github.com/torognes/vsearch), SSU-ALIGN 0.1 [44], and FastTree [45], as well as custom analytic software (source code at https://github.com/caporaso-lab/autism-fmt1) being prepared for release in QIIME 2. Sequence quality control and demultiplexing using QIIME’s split_libraries_fastq.py with default parameters was performed as described in Bokulich et al. [46] on a per-run basis. The sequences were combined across runs by merging the resulting files using the cat Unix command, and sequences were clustered into operational taxonomic units (OTUs) at sequence similarities of 100 and 97%. One-hundred percent OTUs were computed using a pipeline designed for this study. First, sequences were clustered into 100% OTUs with VSEARCH, and the resulting data were loaded into a BIOM table using the biom from-uc command. OTUs that occurred in only one sample were filtered from the table for computational efficiency. OTU representative sequences were aligned with ssu-align, and high entropy positions were filtered with ssu-mask. A phylogenetic tree of representative sequences was built using FastTreeMP for use in phylogenetic diversity analyses, and representative sequences were taxonomically annotated using QIIME’s RDP Classifier wrapper against the Greengenes 13_5 reference database. After filtering OTUs that were observed in only a single sample, a median of 28,486 sequences per sample was observed. Alpha and beta diversity analyses were performed using QIIME’s core_diversity_analyses.py, at rarefaction depths of 5721 (to retain as many samples as possible) and 10,000 to confirm that the results were similar with more sequences per sample. In a parallel analysis, OTUs were clustered at 97% similarity using QIIME’s pick_open_reference_otus.py with the Greengenes 13_5 reference database and default parameters. Engraftment analyses were performed by using custom software that is provided in the GitHub repository referenced above. Statistics were performed using scipy 0.17.0, visualizations were created with seaborn 0.6.0, and all analyses were performed using Project Jupyter (notebook version 4.0.6).

### Isolation and sequencing of viral DNA

Viral DNA was isolated from stool samples as previously described by Minot et al. [47] with slight modifications. Briefly, 0.5 g of stool was resuspended into 40 mL of SM buffer, spun down at 4000 rpm for 30 min, and filtered the supernatant at 0.2 μm. The filtrate was ultra-centrifuged through a CsCl step gradient as detailed in Thurber et al. [48]. To target dsDNA bacteriophages, the 1.35–1.5 g/mL fraction was collected from the CsCl column and was treated with chloroform and then with DNase I (100 U/mL) followed by the addition of 0.1 M EDTA and 0.1 M EGTA to halt enzyme activity as described [49]. Viral DNA was then extracted using the DNeasy Blood & Tissue Kit. Following DNA extraction, the sequencing libraries were prepared using the NexteraXT kit with two minor changes. During the library preparation, input DNA was PCR amplified with 18–25 cycles. When input DNA concentrations were low, the buffer ATM was added at a 1:10 dilution. Sequencing was carried out on a MiSeq v3 2 × 300 at one sixth of a lane per sequencing library.

### Virome bioinformatics

The quality control was performed on sequence reads using Trimmomatic [50] to remove adaptors, trim low-quality ends of reads (reads were cut as soon as the base quality dropped below 20 on a 4 bp window), and discard short reads (< 50 bp). Then, the reads were assembled from each sample using Idba_ud [51] with kmer size varying from 20 to 100 by increment of 10. The assembled contigs were screened with VirSorter [52] to identify and remove all microbial genomes sequences (i.e., all contigs >10 kb and not detected as viral by VirSorter in “virome decontamination” mode). Then, a non-redundant dataset of viral contigs was generated by clustering all viral contigs with Cd-hit [53] using the thresholds previously established (95% ANI on 80% of the shortest sequence) [54, 55]. This resulted in 4759 non-redundant viral sequences longer than 10 kb.

### Analyses of viral populations

To determine the viral population relative abundances in the initial samples, the QC reads were mapped back to this non-redundant contigs database with bowtie2 (option—non-deterministic and non-sensitive, default otherwise) [56]. A contig was considered as detected in a sample if covered by reads on more than 75% of its length, and its abundance was computed as the contig average coverage (number of base pairs mapped to the contig divided by contig length) normalized by the total number of base pairs sequenced in the metagenome [56]. The diversity indices, Shannon’s H′ and Peilou’s J, and Bray-Curtis distances were calculated by using the vegan package [57] in R version 3.2.3 [58]. Bray-Curtis distances were statistically ordinated using the nonmetric multidimensional scaling (NMDS) and then evaluated the influence of the metadata on sample ordination using the “envfit” function with a total of 9999 permutations in the vegan package. Engraftment analyses were performed by using custom Perl scripts. The scripts can be found in the project’s GitHub repository. Viral genes for each viral population were predicted using Prodigal (https://github.com/hyattpd/prodigal/releases/). A blastx for all identified viral genes was performed against the Viral Protein RefSeq to obtain the top three hits with a bitscore of > 50. The familial taxonomy was then obtained for the three hits for each protein. If more than two of the hits had the same familial taxonomy, the viral protein was then assigned that taxonomy. To assign viral taxonomy to the whole viral contig, > 50% of the genes within the contig had to have the same familial taxonomy. To determine if a viral population was similar to the core viral dataset in Manrique et al. [59], the core contigs genomes were obtained from Manrique et al. and used as a blast database. A blastn of the 1651 viral populations in this dataset was performed against the core 23 phage contigs. If a populations had a blastn alignment length of > 500 bp to one the 23 core gut phage contigs at a percent identify greater than 75%, it was considered related to the core 23 phage contigs.

### Code availability

All commands that were applied for the microbiome analyses are provided in the GitHub repository available at http://github.com/caporaso-lab/autism-fmt1 to facilitate reproducibility of these bioinformatics methods.

### Statistical analysis

Statistical analysis was not utilized to predetermine sample size, since the effect size was unknown. Instead, the study was designed based on our previous research in which statistically significant differences within a similar sample size were detected [21]. The previous study was a case-control comparison that did not include an intervention, and so similar or larger differences were assumed to appear as a result of treatment. Since the sample size is still relatively small, and the data are assumed as non-normally distributed, nonparametric analyses were performed, including the Mann-Whitney *U* test, Wilcoxon signed-rank test, and Spearman’s correlation test. All *p* values reported in the study were from two-tailed tests, except the hypothesis on low fiber consumption and low microbial diversity in children with ASD at baseline. *p* values lower than 0.05 were accepted as significant in clinical data analysis. All *p* values for bacterial microbiome analyses were corrected using the Benjamini-Hochberg false discovery rate correction, and the resulting corrected values were referred to as *q* values. *q* values lower than 0.05 were accepted as significant. For some previously hypothesized beneficial bacteria (*Bifidobacterium* and *Prevotella*), *q* values were not significant, but they were considered to be suggestive of statistical significance (*q* values less than 0.1 but greater than 0.05). Statistical significance of variance is reported as indicated per experiment in figure legends. All center values in the box plots are median. The top and bottom edges of the box are of the 75th and 25th percentiles of the sample. *p* values for the phageome analyses are permutation *p* values calculated from 9999 randomized permutations, with *p* values lower than 0.05 accepted as significant.

## Results and discussion

### Subject characteristics

Eighteen children with ASD each from a different family and 20 neurotypical children from 13 families (6 families had 1 neurotypical participant and 7 families had 2 neurotypical participants) were enrolled in the study reported here. All ASD participants completed the 18-week treatment study (neurotypical children were not treated). Neurotypical children had no first-degree relatives of individuals with ASD. Participants in both groups were of similar age, gender distribution, and body mass index (BMI), but the ASD group had more individuals that were delivered by C-section, used non-standard formula during infancy, and had food allergies and eczema (Additional file 1: Table S1). Children with ASD had marginally lower fiber consumption (one-tailed Mann-Whitney *U* test, *p* = 0.07), and their mothers also had significantly lower fiber consumption compared with mothers of neurotypical children (two-tailed Mann-Whitney *U* test, *p* < 0.01). Children with ASD were breastfed significantly shorter time than neurotypical children (two-tailed Mann-Whitney *U* test, *p* < 0.05). Consumptions on carbohydrate, fat, protein, and calorie were comparable between children with ASD and neurotypical children (Additional file 1: Table S1). Other larger studies reported that children with ASD had more antibiotics administered during the first few years of life [11], but this ASD group reported a comparable number of antibiotic administrations to the control group during the first 4 years of life (Additional file 1: Table S1). Children with ASD who had moderate or severe GI problems were recruited, which reflected higher GSRS scores in the ASD group than the control group. A summary on participants’ characteristics and their medical and diet history is listed in Additional file 2: Dataset S1.

### GI and ASD evaluations

**Fig. 2:**
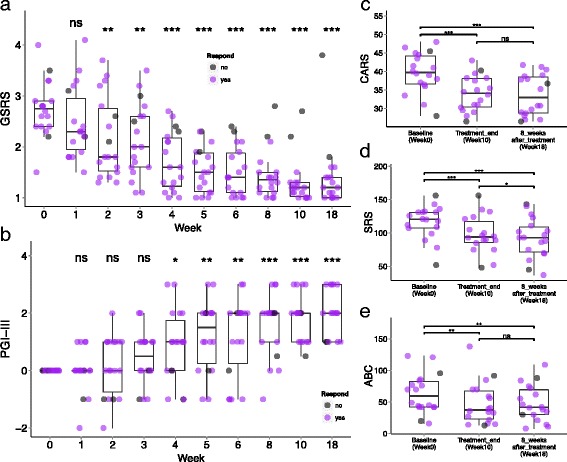
GI- and ASD-related symptoms of 18 children with ASD. Children were treated with MTT for 10 weeks, with a single follow-up evaluation 8 weeks after treatment ended. **a** GSRS scores vs. time. GSRS is scored on a Likert scale from 1 (no symptoms) to 7 (very severe discomfort). **b** Changes in PGI-III scores (overall autism/related symptoms). PGI-III is scored from −3 (much worse), −2 (worse), −1 (slightly worse), 0 (no change), 1 (slightly better), 2 (better) to 3 (much better) compared to baseline. **c** CARS assessment at pre-treatment, post-treatment, and 8 weeks post-treatment. **d** Total SRS score at pre-treatment, post-treatment, and 8 weeks post-treatment. **e** Total ABC score at pre-treatment, post-treatment, and 8 weeks post-treatment. The data points represent 18 individual participants, and some data points overlap in the box plot. *Asterisks* (at the *top of the box plot*) indicate whether individuals (at each time points) have significantly decreased since pre-treatment (week 0). *ns* indicates not significant, *single asterisk* indicates *p* < 0.05, *double asterisks* indicate *p* < 0.01, *triple asterisks* indicate *p* < 0.001 (two-tailed Wilcoxon signed-rank test). Two participants who had less than 50% improvement in GSRS scores are defined as non-responders and color-coded in *grey*

Substantial changes in GI and ASD symptoms were observed. GI symptoms, as assessed by the GSRS, significantly improved for abdominal pain, indigestion, diarrhea, and constipation (Fig. 2a and Additional file 3: Figure S1a). The average GSRS score dropped 82% from the beginning to end of the treatment and remained improved (77% decrease from baseline) even 8 weeks after treatment stopped (two-tailed Wilcoxon signed-rank test, *p* < 0.001). Only two out of 18 children with ASD (11%) achieved less than 50% reduction in the average GSRS, the cutoff for improvement, and were designated as non-responders. Similarly, the DSR showed significant decreases in the number of days with abnormal or no stools (two-tailed Wilcoxon signed-rank test, *p* = 0.002), and those improvements were maintained after 8 weeks of no treatment (Additional file 1: Table S2 and Additional file 3: Figure S1b).

Beyond these GI improvements, ASD-related behavior also improved following MTT. The PGI-II assessment, which evaluates 17 ASD-related symptoms, revealed significant improvement during treatment and no reversion 8 weeks after treatment ended (Fig. 2b). Further, a significant negative correlation between change in GSRS and PGI-III (Spearman’s correlation test showed *r* = −0.59 and *p* < 0.001, Additional file 3: Figure S2) suggests that GI symptoms worsen directly with ASD behaviors, and that these can be altered via MTT. The scores on CARS, which rates core ASD symptoms, decreased by 22% from beginning to end of the treatment and 24% (relative to baseline) after 8 weeks of no treatment (Wilcoxon signed-rank test, *p* < 0.001, Fig. 2c). Children with ASD saw improvement in their scores in the SRS, which assesses social skill deficits, and the ABC, which evaluates irritability, hyperactivity, lethargy, stereotypy, and aberrant speech (Fig. 2d, e). The VABS-II scoring, which evaluates adaptive behaviors such as communication, daily living skills, and socialization, found that the average developmental age increased by 1.4 years (*p* < 0.001) and across all sub-domain areas (Additional file 3: Figure S3) during MTT, though the final VABS-II age equivalent was still lower than their chronological age. Finally, MTT appears to be beneficial for children ages 7–16 years old (no significant correlations between age and GSRS or CARS improvement), and there was no significant difference in clinical outcomes between those who received the initial SHGM dose orally or rectally.

The MTT treatments were generally well-tolerated, with only temporary adverse effects (primarily mild to moderate hyperactivity and tantrums/aggression) at the beginning of vancomycin treatment, no major changes in blood chemistry or long-term adverse effects were noted. Detailed information is provided in Additional file 4. The improvements in GI and ASD symptoms are consistent with a previous 8-week trial of the use of vancomycin for treating children with ASD [22], but a key difference is that in the previous study, benefits were lost within a few weeks of stopping vancomycin therapy (despite the use of standard probiotics in some children), whereas in this study, the benefits continued for at least 8 weeks. It is also relevant to note that GI and ASD symptoms slowly improved over the 10-week MTT treatment and 8-week observation period, since this observation is very different from FMT treatment for *C. difficile*, where a single dose generally leads to recovery within a few days [60]. Thus, it appears likely that extended treatment with FMT over many weeks, as done in this study, is necessary to observe these benefits.

### Bacterial changes after MTT

**Fig. 3:**
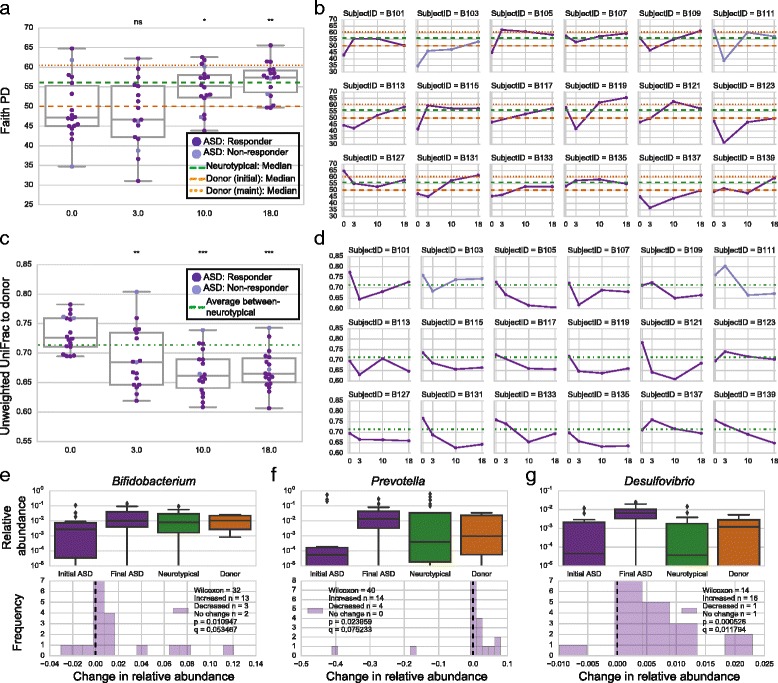
Stool microbiota changes with fecal microbiota transplant. **a** Changes in Faith’s phylogenetic diversity (PD) in the microbiota of 18 children with ASD as measured from stool samples. *Orange lines* indicate median PD of the donor samples (*dashed line* represents initial donor samples (*n* = 5), and *dotted line* represents maintenance dose samples (*n* = 2)), and *green line* indicates median PD of 20 neurotypical controls at week 0. *ns* indicates not significant, *single asterisk* indicates *q* < 0.05, *double asterisks* indicate *q* < 0.01, *triple asterisks* indicate *q* < 0.001 (two-tailed Wilcoxon signed-rank test comparing weeks 3, 10, and 18 to week 0 values). **b** Change in Faith’s PD tracked on a per individual basis for all MTT recipients. Most individuals experienced an increase in gut microbiota PD. **c** Unweighted UniFrac distances between ASD gut microbiota and most relevant donor sample (initial donor sample at weeks 0 and 3, most recent maintenance dose sample at weeks 10 and 18). *Green line* indicates the median interpersonal variation between neurotypical controls and illustrates that prior to treatment the difference in gut microbiota composition between MTT recipients and donors was on the order of normal interpersonal variation. Following treatment, the MTT recipients were more similar to donors than normal interpersonal variation. Statistics are the same as those used in **a. d** Distances between ASD gut microbiota and donor sample on a per individual basis. Most individuals became more similar to the donor over the study period. **e–g** Box plots illustrating relative abundances of three genera, *Bifidobacterium, Prevotella*, and *Desulfovibrio*, in the gut microbiota by group (*top*; log scale), and changes in relative abundances at week 18 in the ASD group (*bottom*). All *p* values were corrected using the Benjamini-Hochberg false discovery rate correction to create *q* values. Analogous plots based on different diversity metrics are presented as supplementary figures

Given these strong clinical responses to MTT, changes in bacterial and phage diversity in gut samples over time as well as correlations to clinical data were sought (details in “Methods” section). Based on the phylogenetic diversity (PD) index [61], gut bacteria were significantly less diverse in children with ASD than neurotypical controls at baseline (Fig. 3a; one-tailed Mann-Whitney *U* test, *p* = 0.027), which is consistent with prior work [21]. After major SHGM intervention at week 3, an increase in diversity compared with baseline was not observed, suggesting that initial SHGM restored diversity that was reduced by the vancomycin treatment. Without a control arm including individuals who are only treated with vancomycin, we cannot absolutely attribute this recovery to the SHGM, and a follow-up study with this hypothesis is warranted. At the end of treatment, however, bacterial diversity increased in children with ASD (Fig. 3a; two-tailed Wilcoxon signed-rank, *p* < 0.05 and *p* = 0.001, respectively), and remained higher than baseline 8 weeks after treatment stopped, such that median richness at week 18 was statistically indistinguishable between the ASD and control groups (Fig. 3a; two-tailed Mann-Whitney *U* test, *p* = 0.78). This increase was observed in 16 out of 18 individuals including one of the two non-responders (subjects whose GI symptoms improved less than 50% on the GSRS) (Fig. 3b). Similar results of initial low diversity, followed by an increase to those in neurotypical children after MTT, were also observed using a non-phylogenetic metric, *Observed OTUs* (Additional file 3: Figure S4). Higher gut bacterial diversity and richness are commonly associated with healthy status, presumably due to resilience afforded by higher functional redundancy [62].

Importantly, the donor bacterial community was at least partially engrafted in the recipient gut, consistent with earlier work [63] and a recent study of the efficacy of FMT [64]. Specifically, the unweighted UniFrac distance (i.e., a qualitative measure of the dissimilarity of a pair of microbial communities based on shared OTUs) between the host gut and their most recent donor sample significantly decreased over time (Fig. 3c; two-tailed Mann-Whitney *U* test *p* < 0.01 at 3 weeks and *p* < 0.001 at 10 and 18 weeks) and remained more similar to the donor’s bacterial community 8 weeks after treatment stopped. By the end of the treatment (week 10) and 8 weeks after the treatment stopped (week 18), the distance between the recipient and the donor bacterial community was less than normal interpersonal bacterial community variation (in this case, defined by variation between the neurotypical controls) (Fig. 3c). These signatures of engraftment suggest that MTT overcame “colonization resistance” [65].

Specific genera that significantly changed in their relative abundances with treatment included *Bifidobacterium*, *Prevotella*, and *Desulfovibrio* (Fig. 3e–g and Additional file 5: Dataset S2). *Bifidobacterium* was reported to be underrepresented in children with ASD [7, 21, 66], also observed in this study at baseline (two-tailed Mann-Whitney *U* test *p* < 0.05), but following MTT, the relative abundance of *Bifidobacterium* significantly increased fourfold and became comparable to its relative abundance in neurotypical children (Fig. 3e). This suggests strong engraftment by these microbes in particular. Additionally, relative abundances of *Prevotella* and *Desulfovibrio* significantly increased after MTT from baseline to 8 weeks following treatment (Fig. 3f, g). Initially, the relative abundance of *Prevotella* was comparable between neurotypical children and children with ASD at baseline, which was not consistent with our previous cohort study with 20 neurotypical children and 19 children with ASD [21]. However, the increase in the relative abundance of *Prevotella* after MTT is consistent with their potentially beneficial role in the gut of children with ASD. The increased relative abundance of *Desulfovibrio* is intriguing, since their role in the human gut has been controversially proposed as either commensal [21] or detrimental [18, 67]. Both *Prevotella* and *Desulfovibrio* were on average more abundant in MTT recipients following treatment than in the donor samples, illustrating that the transferred microbiota changes the gut environment in a way that is more hospitable to recruit new commensal bacteria. Taken together, these data suggest that MTT successfully shifts the ASD bacterial community toward that of age/gender-matched healthy controls and to that of their donors.

### Phage community changes after MTT

**Fig. 4:**
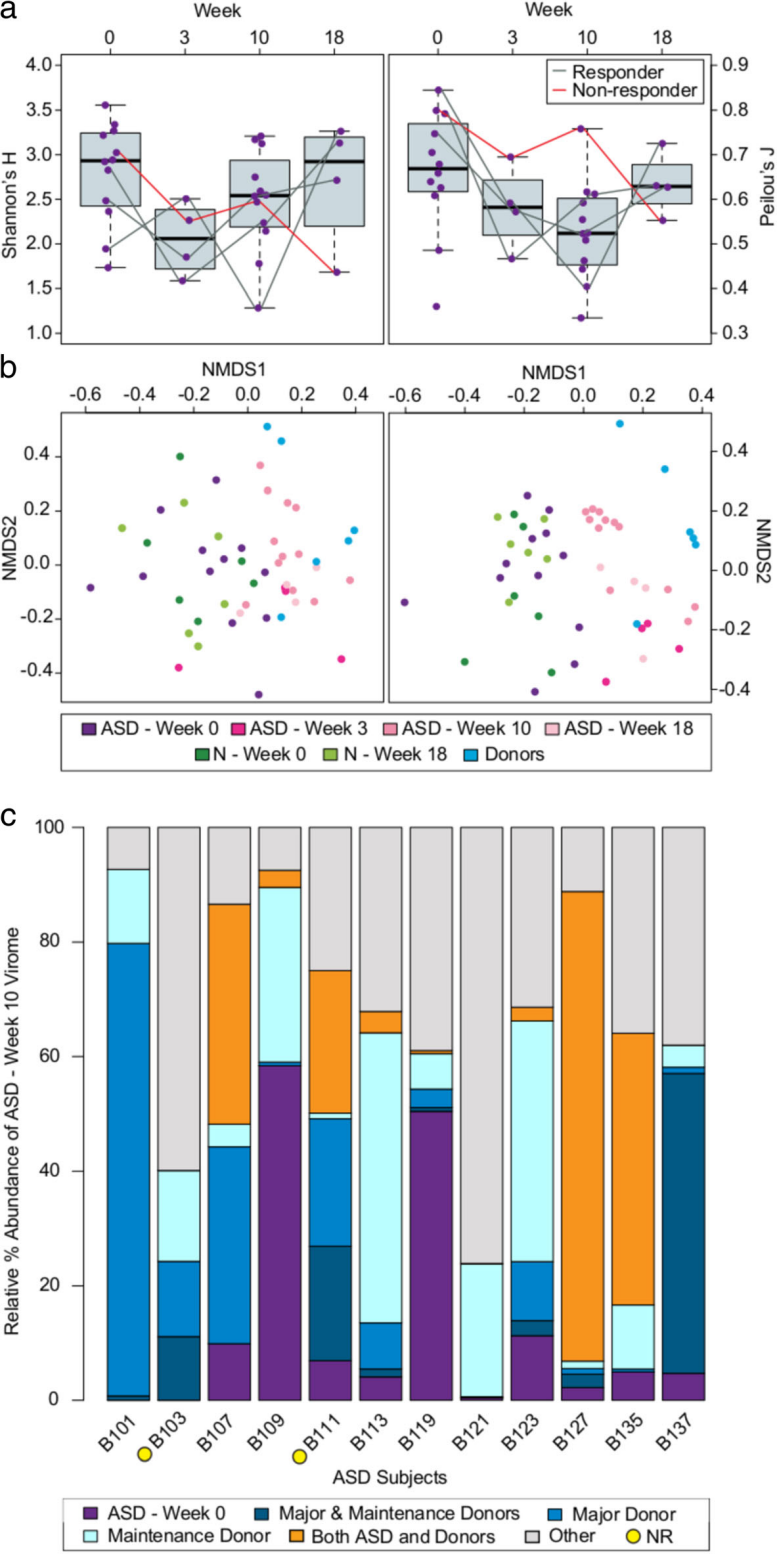
Stool virome change with fecal microbiota transplant. **a** Diversity indices, Shannon’s H′ (a measure of biodiversity and richness; *left*) and Peilou’s J (a measure of evenness; *right*), of the ASD participants. Fecal samples were collected at all four time points for 4 out of the 12 ASD subjects where the bacteriophage communities were assessed. The responders (indicated by a *grey line*) rebounded in biodiversity, richness, and evenness following MTT. In contrast, the non-responder (indicated by a *red line*) did not recover. **b** Nonmetric multidimensional scaling of Bray-Curtis dissimilarity (*right*; 2D stress = 0.2467) and Jaccard (*left*; 2D stress = 0.2212) distances reveal that ASD gut bacteriophage communities are more similar to donor gut bacteriophage communities following both the high and lower SHGM doses. **c** Analyses of ASD virome composition at week 10 shows engraftment of donor bacteriophage populations across all ASD subjects. In > 80% of the subjects, the starting (week 0) bacteriophage populations make up < 20% of the virome at week 10. NR stands for non-responder

Since phage analysis is extremely intensive and costly, and this was a pilot project, an exploratory evaluation of only a subset of the stool samples mainly focusing on ASD samples from week 0 and 10 was conducted to determine their phage content. Sample selection was conducted prior to the availability of bacterial 16S rRNA gene sequencing data, so focus turned to ASD week 10 samples rather than week 18 samples in case the effects of MTT were not detectable following the termination of treatment. Of the detected phage populations, most (95.64%) were unknown, but the rest were part of the order *Caudovirales*, with 2.97% assigned to the family *Siphoviridae*, 0.73% to *Myoviridae*, and 0.67% to *Podoviridae* (Additional file 3: Figure S5). In contrast to the gut bacteria, phage richness and evenness did not significantly change following MTT given the timeframe of this study (Fig. 4a). This is not surprising given that, at the population level, phage communities are reliant on their host communities and, thus, significant changes in phage diversity can lag behind bacterial community changes [68]. Nonetheless, a number of metrics suggested phage communities also responded to MTT as follows. First, four individuals were tracked longitudinally from week 0 to week 18—three who clinically responded to MTT and one non-responder. In all cases, the phage diversity initially decreased (likely due to the effect of vancomycin treatment on their host) and then recovered only for the three responders (Fig. 4a). Second, community dissimilarity metrics revealed that MTT resulted in phage communities of children with ASD becoming more similar to those from the donor (Fig. 4b). Permutation-based fitting of subject variables to Bray-Curtis and Jaccard NMDS plots uncovered significant clustering based on subject type (e.g., ASD, neutrotypic (N) and donor; *r* ^2^ ≥ 0.2120, *p* ≤ 0.0001, 9999 permutations) and among ASD subjects based on treatment stage (*r* ^2^ ≥ 0.4021, *p* ≤ 0.0002, 9999 permutations) and high (*r* ^2^ ≥ 0.2066, *p* ≤ 0.0149, 9999 permutations) and low (*r* ^2^ ≥ 0.1851, *p* ≤ 0.0023, 9999 permutations) SHGM doses. Finally, based on comparisons between starting phage communities and week 10 communities, phage populations from the donor were found engrafted across all ASD subjects, while the abundance of phage populations originally in their pre-MTT virome were completely eliminated or decreased (Fig. 4c).

While the role of phages in the gut is largely understudied in comparison to the role of bacteria, this study and recent research has begun to uncover the potential role of phages in the gut. In healthy individuals, the gut virome is highly stable over time [23, 47, 69], with some phage populations hypothesized to provide a non-host-derived protective barrier to invading bacterial pathogens [70, 71]. While there is high inter-individual variation [47], recent analyses have identified a distinct subset of phages that are found across the majority of healthy individuals [59]. These “healthy” phage populations represent < 5% of the phage population identified in our study (Additional file 3: Figure S5). In individuals with gastrointestinal disease (i.e., ulcerative colitis and Crohn’s disease), these phage populations represent a significantly smaller percentage of the gut phage community [59]. This shift in viral community structure is hypothesized to allow potentially harmful bacteria and viruses to proliferate through phage-mediated dysbiosis, whereby perturbations to the healthy gut phage community leads to increased abundances of phages and selected reduction in bacterial species [70–72].

Studies looking at the effects of perturbations in the gut from Crohn’s disease in humans [73] and from diet in mice [74] show this response with increased diversity of phage communities paired with decreased diversity of the bacterial community. In this study that looks at reversing the negative responses to gut perturbations caused by ASD, no significant changes were observed in the diversity of the phage community, but an altered phage community paired with an increase in the diversity of the bacterial community. This suggests that MTT may be able to reverse phage-mediated dysbiosis of the ASD gut, though further study is necessary to test this assertion.

### Study limitations and recommendations

Although study observations are noteworthy, the current open-label trial is not placebo controlled, blinded, or randomized. Here, we list some limitations and how they should be addressed in a follow-up blinded trial with a placebo control arm. First, this exploratory study only looked at the consequences of the combined treatment of MTT. Follow-up studies are needed to determine whether MTT efficacy in our study results solely from vancomycin, MoviPrep, SHGM, Prilosec, or a combination of these four factors. Sandler et al. [22] reported a temporal efficacy of vancomycin treatment in GI and ASD symptoms, but this study involved a small number of participants who were younger than 7 years old. Previous studies have shown that vancomycin [75] and proton pump inhibitors [76] significantly alter gut microbiota. Further studies are, however, also essential in order to clarify how each factor in MTT contributes on changes in gut microbiota in the context of ASD. Second, in this study, participants had a range of GI issues, including constipation, diarrhea, and alternating diarrhea/constipation. Larger studies in future would allow us to look at those groups separately. Alternatively, a more homogeneous cohort (e.g., children with shared GI issues and ASD etiologies and similar ages) would allow for better disentanglement of the signal from inter-individual variation from FMT efficacy, since inter-individual gut bacterial [77] and viral [47] community variation is high. Third, since this was an open-label study, the effect on GI and ASD symptoms are likely to be subject to placebo effects and should be cautiously interpreted and viewed as preliminary. Fourth, a clinical trial with extended longer observation period after treatment would help determining long-term safety and possible benefits. Lastly, a larger sample size will be essential to clarify associations with other variables, such as the efficacy of oral versus rectal administration of the SHGM.

Further, in follow-up studies, continued use of GI and behavior assessments to carefully track changes in ASD severities, with some additional modifications, is recommended. In this study, the GSRS, SRS, ABC, PGI-III, and VABS-II assessments are reported by parents/guardians, consulting with subjects verbally if the subjects were adolescents. Previous ASD clinical studies have reported disagreement between parent report and that of a pediatric gastroenterologist in terms of the specific GI symptoms and diagnoses, for some metrics (e.g., GI assessment instrument—QPGS-Rome III [78]). As a result, clinical expertise, in addition to parent/subject reports, could provide more reliable and independent assessments.

## Conclusions

Together, these findings suggest that MTT is safe and well-tolerated in children with ASD ages 7–16 years. MTT led to significant improvements in both GI- and ASD-related symptoms, and the improvements were sustained at least 8 weeks after treatment. Coincident with these clinical improvements, both microbiota and phage from the donors appear to have engrafted, at least partially, in the recipients. This shifted gut microbiota of children with ASD toward that of neurotypical children is consistent with the hypothesis that gut microbiota may be at least partially responsible for GI and ASD symptoms. While this study was an open-label trial that is subject to placebo effects, these results are promising and provide a crucial step for understanding the connection between the microbiome and ASD. A randomized, double-blind, placebo-controlled study is the next step to investigate the value of MTT in treating children with ASD and GI problems.

## Additional files

Additional file 1: Table S1. Characteristics of study participants and their medical and diet history. All values are median ± median absolute deviation (MAD). *p* values are either by Mann-Whitney *U* test or Fisher’s exact probability test. n.s., not-significant. **Table S2.** Percent days of no stool, stool hardness, and softness based on the daily stool record (DSR) and the Bristol Stool Form Scale (*p* values by two-tailed Wilcoxon signed-rank test). **Table S3.** Adverse effects. (PDF 72 kb)
Additional file 2: Dataset S1. A summary on participants’ characteristics and their medical and diet history. (XLSX 53 kb)
Additional file 3: Figure S1. Subscores of GI- and ASD-related symptoms in 18 children with ASD. **Figure S2.** Correlation between percentage change in GSRS and overall PGI-III scores (based on the data shown in Fig. 2a, b) for the 18 weeks of the study. **Figure S3.** Vineland developmental age (in years) for individual subscales and for the average of all subscales, measured at baseline and at the end of observation 4 months later. **Figure S4.** Stool microbiota changes in community richness with fecal microbiota transplant. **Figure S5.** Gut phageome taxonomy is still mostly unknown. **Figure S6.** Subscores of the PGI-III at end of treatment (week 10). **Figure S7.** Microbiota changes with fecal microbiota transplant based on swab samples (analog of Fig 3a–d). **Figure S8.** Stool microbiota changes with fecal microbiota transplant. **Figure S9.** Engraftment plots with four diversity metrics (stool samples). Figure S10. Stool microbiota changes with fecal microbiota transplant. (PDF 1964 kb)
Additional file 4 Supplementary text. (PDF 123 kb)
Additional file 5: Dataset S2. Taxonomy changes in genus level after MTT. (XLSX 32 kb)

## Abbreviations

ABC: Aberrant Behavior Checklist
ADHD: Attention-deficit hyperactivity disorder
ADI-R: Autism Diagnostic Interview-Revised
ASD: Autism spectrum disorder
BMI: Body mass index
CARS: Childhood Autism Rating Scale
DSR: Daily stool records
FDA: Food and Drug Administration
FMT: Fecal microbiota transplant
GI: Gastrointestinal
GMP: Good manufacturing processes
GSRS: Gastrointestinal Symptom Rating Scale
MTT: Microbiota transfer therapy
NMDS: Nonmetric multidimensional scaling
OTUs: Operational taxonomic units
PGI-III: Parent Global Impressions–III
QIIME: Quantitative Insights into Microbial Ecology
SHGM: Standardized human gut microbiota
SRS: Social Responsiveness Scale
VABS-II: Vineland Adaptive Behavior Scale II
