# Supplementary material for: Microbiota Transfer Therapy alters gut ecosystem and improves gastrointestinal and autism symptoms: an open-label study
Source: Microbiome. 2017 Jan 23;5:10. doi: 10.1186/s40168-016-0225-7 (PMC5264285; doi:10.1186/s40168-016-0225-7)
Supplement: Additional file 1: Table S1. — Characteristics of study participants and their medical and diet history. All values are median ± median absolute deviation (MAD). p values are either by Mann-Whitney U test or Fisher’s exact probability test. n.s., not-significant. Table S2. Percent days of no stool, stool hardness, and softness based on the daily stool record (DSR) and the Bristol Stool Form Scale (p values by two-tailed Wilcoxon signed-rank test). Table S3. Adverse effects. (PDF 72 kb) [file 40168_2016_225_MOESM1_ESM.pdf]

Additional file 1: Table S1. Characteristics of study participants and their medical and diet history. All values are median ± median absolute deviation (MAD). p-values are either by Mann-Whitney U-test[a] or Fisher Exact Probability test[b]. n.s.: not-significant

	Children with ASD	Neurotypical children	P-value (two-tailed)
Total number	18	20	
Male/Female	16/2	18/2	
Age	10.8 ± 1.6	11.4 ± 2.5	n.s.[a]
Body mass index (BMI)	18.2 ± 2.2	17.1 ± 1.1	n.s.[a]
GSRS 4-point scale (sum of all 15 items, minimum score for no symptoms is 15)	28.0 ± 3.3	18.8 ± 3.0	P<0.001[a]
Born by C-Section[1]	61%	15%	P<0.01[b]
Number of months of breastfeeding exclusively (no formula)	3.0 ± 2.8	8.8 ± 6.7	P<0.05[a]
% using non-standard formula (soy or other)[2]	39%	5%	P<0.05[b]
Food allergy (moderate or severe)	56%	5%	P<0.001[b]
Other allergies (moderate or severe)	44%	10%	P=0.25[b]
Eczema	56%	5%	P<0.001[b]
Fiber consumption (grams) - child	9.4 ± 4.2	11.0 ± 2.5	P=0.15[a,c]
Fiber consumption (grams) - mother[3]	6.2 ± 1.3	8.6 ± 1.3	P<0.01[a]
Carbohydrate consumption (grams) - child	108.9 ± 37.8	126.3 ± 30.9	P=0.51[a]
Fat consumption (grams) - child	48.5 ± 10.6	45.0 ± 11.8	P=0.45[a]
Protein consumption (grams) - child	52.4 ± 17.5	47.9 ± 7.9	P=0.75[a]
Total calorie consumption (kcal) - child	1,135.3 ± 316.4	1,014.9 ± 160.9	P=0.89[a]
Oral antibiotic use during first 4 years of life (number of rounds)	2.5 ± 3.6	1.0 ± 3.7	n.s.[a]

[a] P values are from two-tailed Mann-Whitney U-test.

[b] The binary dataset was tested by two-tailed Fisher Exact Probability test.

[c] One-tailed Mann-Whitney U-test P value was 0.07.

[1]1 mother of neurotypcial children did not report the information.
[2]Mothers of 7 neurotypical children did not use any formula.
[3]Mother with Children with ASD (n=12); mothers with neurotypical children (n=13)

Additional file 1: Table S2. Percent days of no stool, stool hardness and softness based on the daily stool record (DSR) and the Bristol Stool Form Scale (P-values by two-tailed Wilcoxon signed-rank test)

				P-value (Baseline vs.)	
	Base line	Treatment end	8 weeks after treatment	Treatment end	8 weeks after treatment
No stool	33%	26%	26%	0.29	0.29
Hard stool (type 1 or 2)	19%	6%	3%	0.07	0.002
Soft/liquid stool (type 6 or 7)	10%	2%	3%	0.03	0.09
Abnormal stool (in total of hard, soft/liquid/ and no stool)	62%	34%	32%	0.002	0.002

Additional file 1: Table S3. Adverse effects.

Adverse effect	% adverse effects
Rash	5% (due to natural orange flavor in vancomycin)
Hyperactivity	39%* (temporary: start of vancomycin only)
Tantrums/aggression	28%* (temporary: start of vancomycin only)
Nausea/vomiting	5% (due to high-dose SHGM)

* The severity of symptoms ranged from mild to moderate.
