# Supplementary material for: Microbiota Transfer Therapy alters gut ecosystem and improves gastrointestinal and autism symptoms: an open-label study
Source: Microbiome. 2017 Jan 23;5:10. doi: 10.1186/s40168-016-0225-7 (PMC5264285; doi:10.1186/s40168-016-0225-7)
Supplement: Additional file 3: Figure S1. — Subscores of GI- and ASD-related symptoms in 18 children with ASD. Figure S2. Correlation between percentage change in GSRS and overall PGI-III scores (based on the data shown in Fig. 2a, b) for the 18 weeks of the study. Figure S3. Vineland developmental age (in years) for individual subscales and for the average of all subscales, measured at baseline and at the end of observation 4 months later. Figure S4. Stool microbiota changes in community richness with fecal microbiota transplant. Figure S5. Gut phageome taxonomy is still mostly unknown. Figure S6. Subscores of the PGI-III at end of treatment (week 10). Figure S7. Microbiota changes with fecal microbiota transplant based on swab samples (analog of Fig 3a–d). Figure S8. Stool microbiota changes with fecal microbiota transplant. Figure S9. Engraftment plots with four diversity metrics (stool samples). Figure S10. Stool microbiota changes with fecal microbiota transplant. (PDF 1964 kb) [file 40168_2016_225_MOESM3_ESM.pdf]

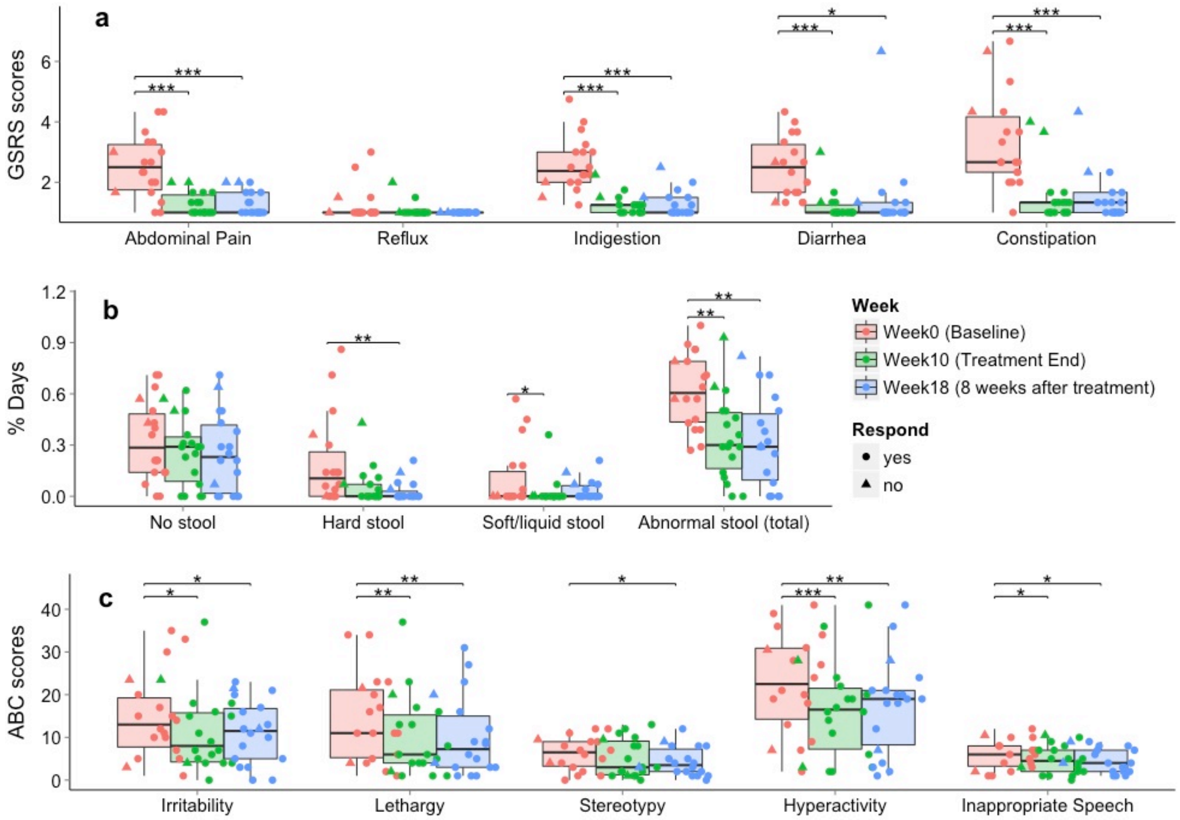

Additional file 3: Figure S1. Subscores of GI- and ASD-related symptoms in 18 children with ASD. a GSRS subscores at baseline, FMT treatment end, and 8 weeks after treatment. **b** Results of daily stool records, averaged over 2 weeks. **c** Subscales of the ABC vs. time. *: p<0.05, **: p<0.01, ***: p<0.001 (Wilcoxon signed-rank test).

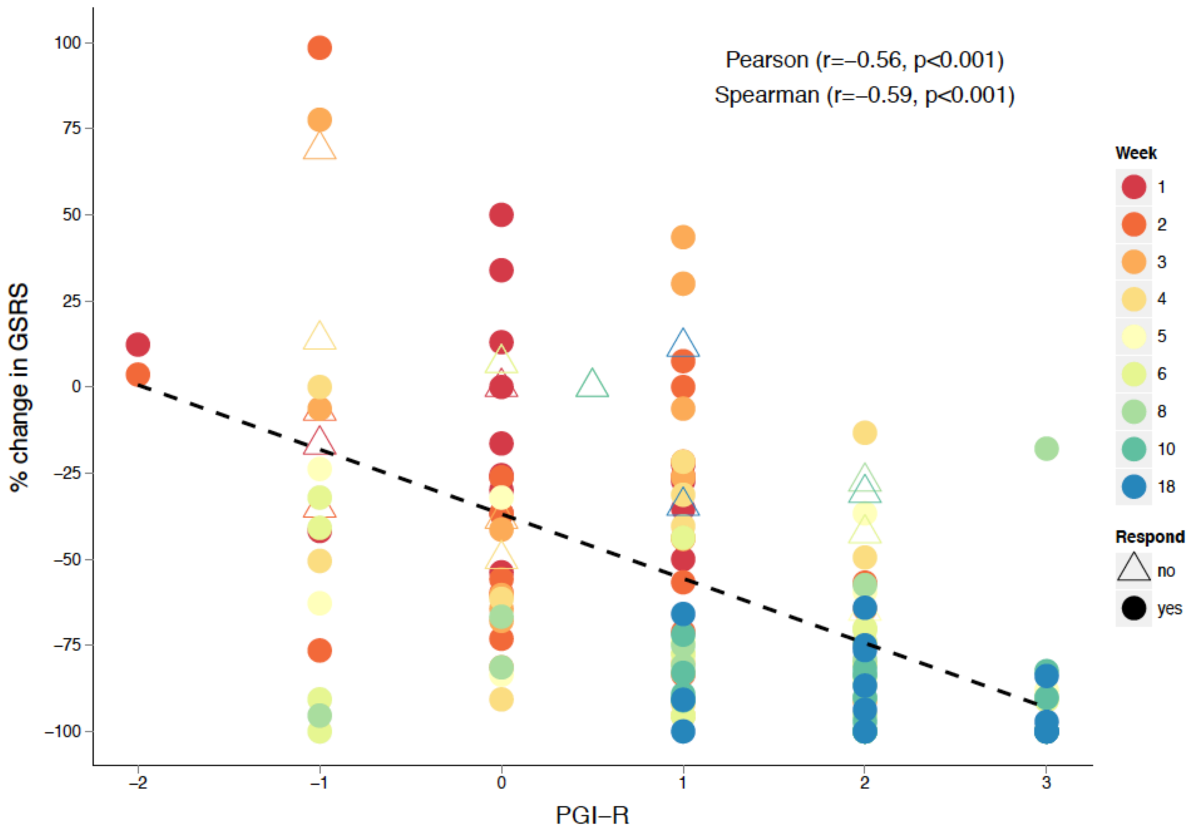

Additional file 3: Figure S2. Correlation between % change in GSRS and overall PGI-III scores (based on the data shown in Fig. 2a, b) for the 18 weeks of the study. The Spearman and Pearson correlation test showed significantly negative correlation (r=-0.59 and -0.56, respectively; p<0.001 for both tests).

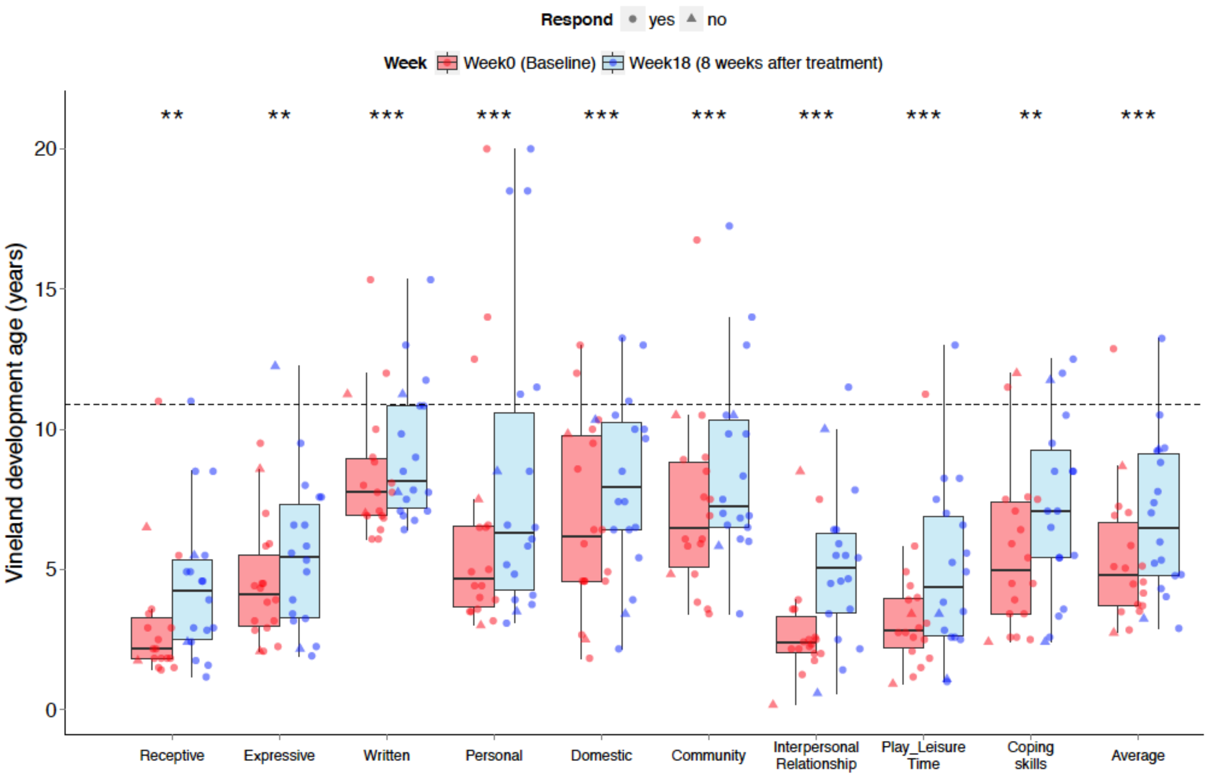

Additional file 3: Figure S3. Vineland Developmental Age (in years) for individual subscales and for the average of all subscales, measured at baseline and at the end of observation 4 months later. Note that the average chronological age was 10.9 years at the start of treatment (showed in a dotted line). As seen here, at baseline there were delays in all areas, especially in the core autism areas of language and social (interpersonal) ability. Subscales are under either communication domain (receptive, expressive, and written), or daily living skills domain (personal, domestic, and community), or socialization domain (Interpersonal relationships, play and leisure Time, and coping skills). *: p<0.05, **: p<0.01, ***: p<0.001 (two-tailed Wilcoxon signed-rank test).

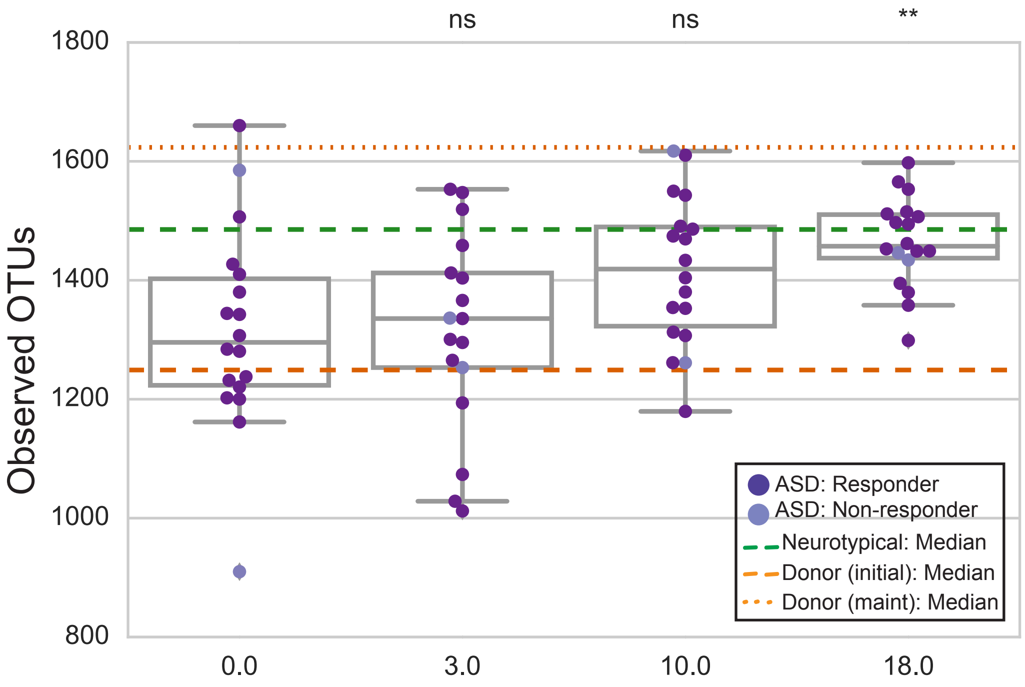

Additional file 3: Figure S4. Stool microbiota changes in community richness with fecal microbiota transplant. Change in community richness, as measured by *Observed OTUs*, a non-phylogenetic diversity metric.

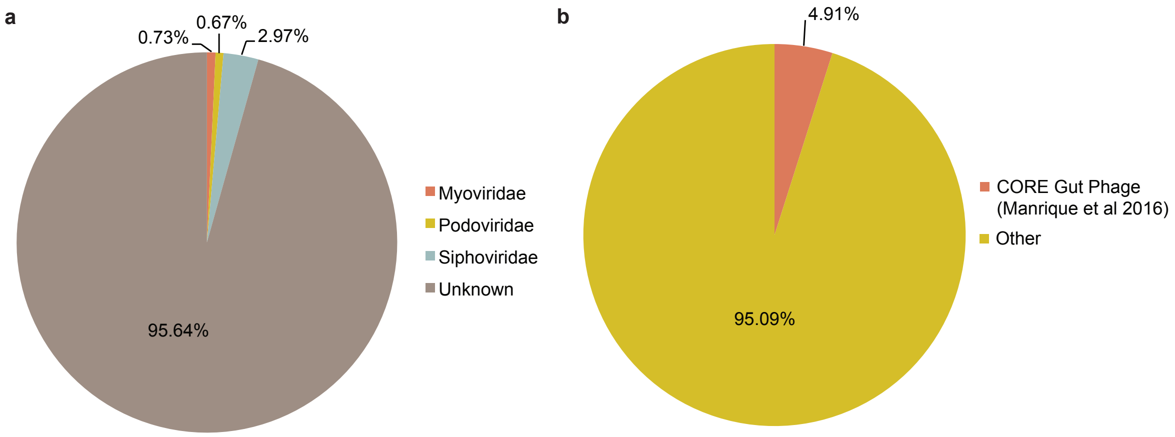

Additional file 3: Figure S5. Gut phageome taxonomy is still mostly unknown. a Viral populations were assigned to a viral family if >50% of the genes in the viral contig had a blastx bit score >50 to the to the same viral family in the Viral Protein RefSeq database. All viral populations that could be assigned to a viral family were members of the Order Caudovirales, with 2.97% assigned to the family Siphoviridae, 0.73% to Myoviridae, and 0.67% to Podoviridae. Most viral populations (95.64%) could not be assigned a familial level taxonomy. **b** Viral populations were considered to related to the 23 core gut phages identified in Manrique et al. [59] if they had a blastn alignment length ≥ 500bp to one the 23 core gut phage contigs at a percent identify greater than 75%. In total, 4.91% of the gut viral populations identified in our study were related to the core phages.

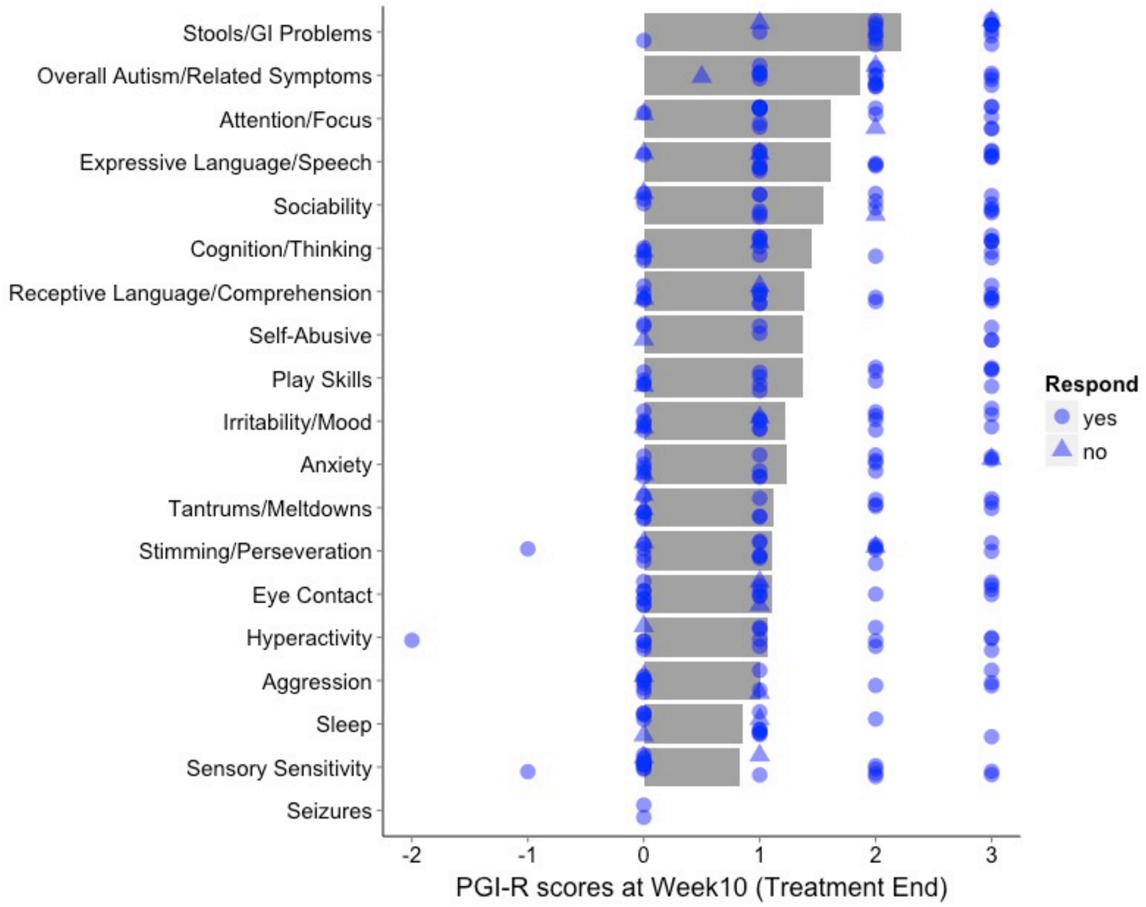

Additional file 3: Figure S6. Subscores of the PGI-III at end of treatment (week 10). The scale goes from 3 (much better) to 2 (better) to 1(slightly better) to 0 (no change) to minus 3 (much worse). Scores were similar after 8 weeks of no treatment (week 18). The data points represent 18 individual participants, and some data points overlap in the box plot. The bar represents mean values of subscores.

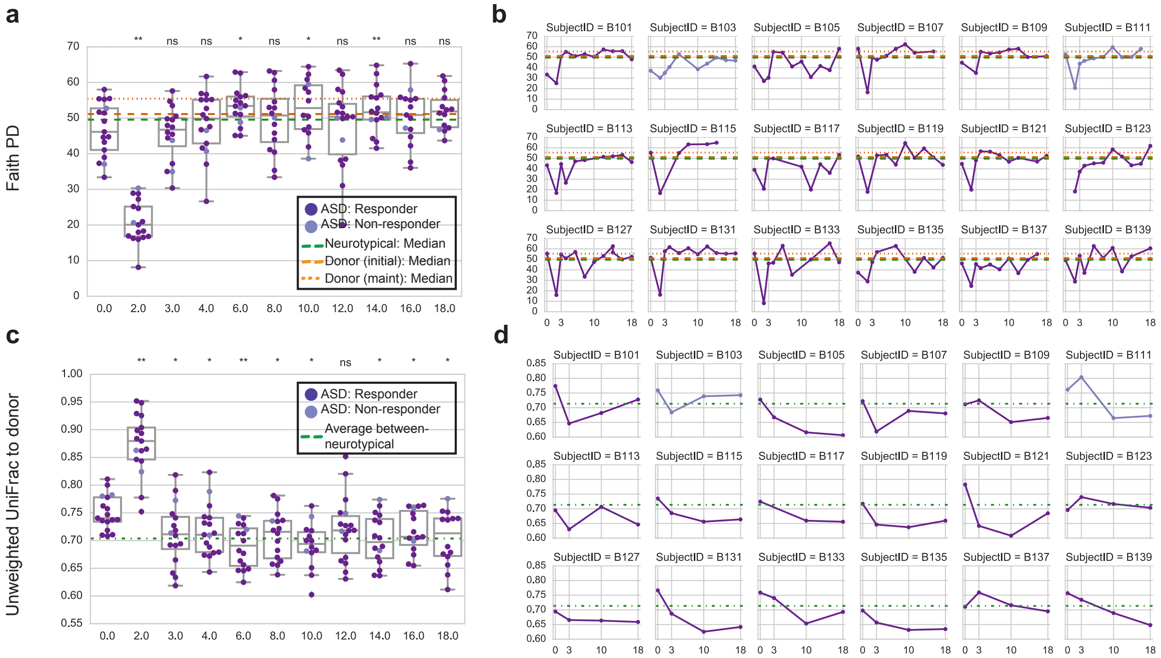

Additional file 3: Figure S7. Microbiota changes with fecal microbiota transplant based on swab samples (Analog of Fig 3. a-d). a Changes in Faith's Phylogenetic Diversity (PD) in the ASD microbiota (n=20). Orange lines indicates median PD of the donor samples (dashed line represents initial donor samples (n=4), and dashed line represents maintenance dose samples (n=2)), and green line indicates median PD of neurotypical controls at week 0 (n=18). **b** Change in Faith PD tracked on a per individual basis for all MTT recipients. **c** Unweighted UniFrac distances between ASD gut microbiota and most relevant donor sample (initial donor sample at weeks 0 and 3, most recent maintenance dose sample at weeks 10 and 18). Green line indicates the median interpersonal variation between neurotypical controls. **d** Distances between ASD gut microbiota and donor sample on a per individual basis.

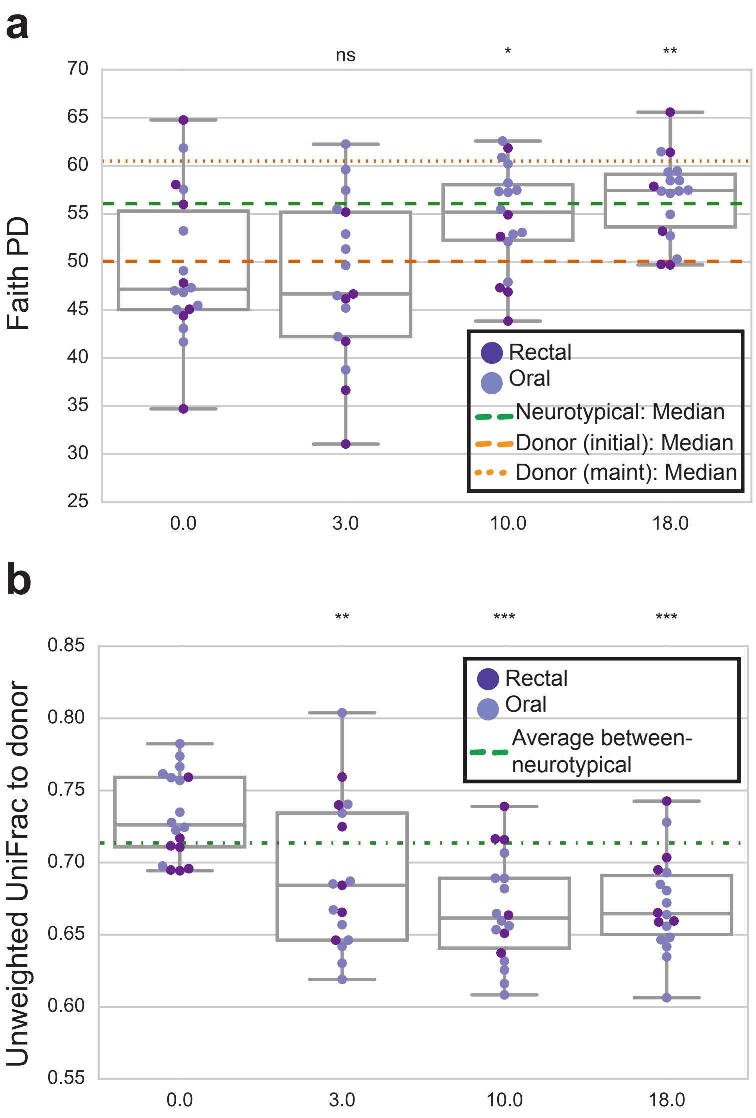

Additional file 3: Figure S8. Stool microbiota changes with fecal microbiota transplant. Analog of Fig. 3a, c, where points are instead colored by whether FMT was given by oral or rectal administration.

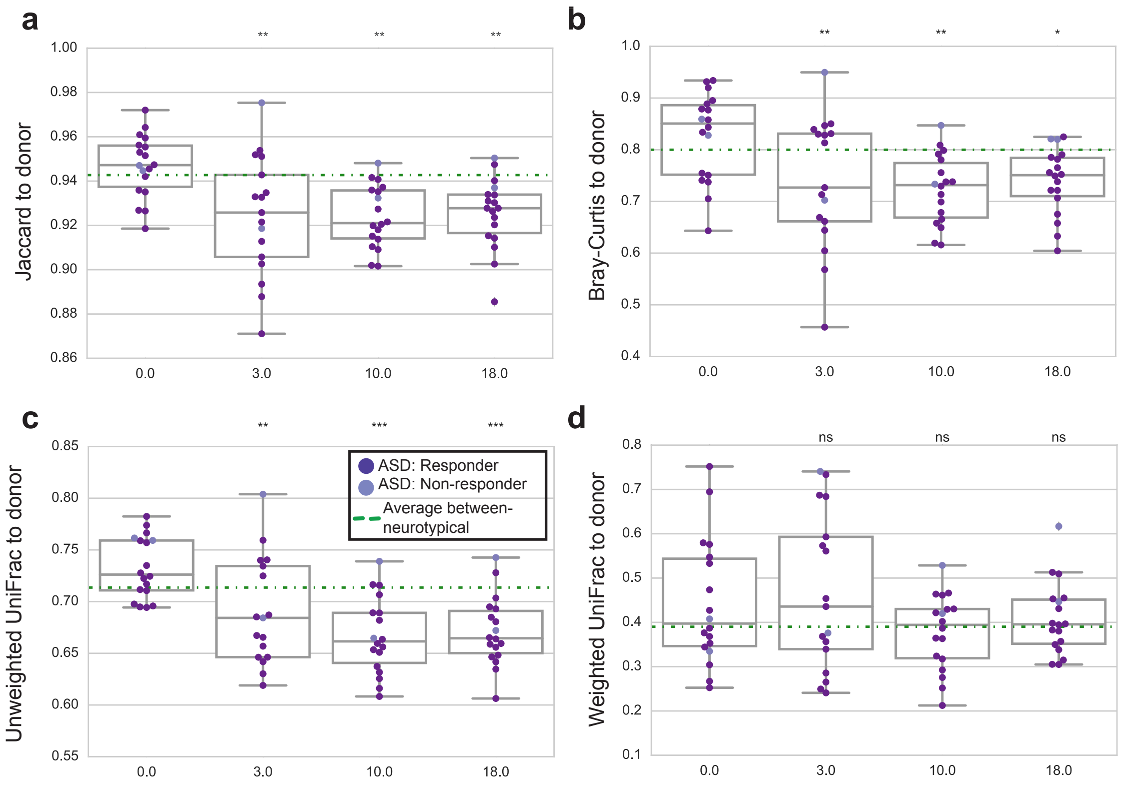

Additional file 3: Figure S9. Engraftment plots with four diversity metrics (Stool samples). A decrease in the distance to donor is an indication that some engraftment has occurred.

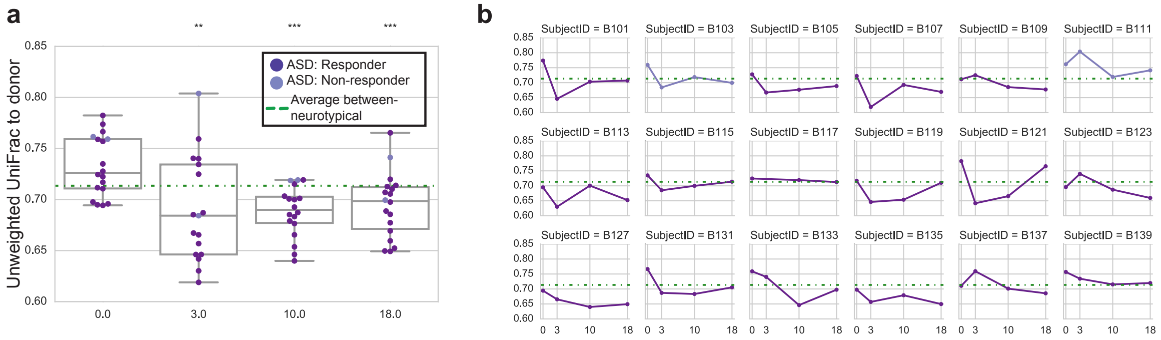

Additional file 3: Figure S10. Stool microbiota changes with fecal microbiota transplant. a Unweighted UniFrac distances between ASD gut microbiota and major initial donor sample. Green line indicates the median interpersonal variation between neurotypical controls and illustrates that prior to treatment the difference in gut microbiota composition between MTT recipients and donors was on the order of normal interpersonal variation. Following treatment, the MTT recipients were more similar to donors than normal interpersonal variation. *: q<0.05, **: q<0.01, ***: q<0.001 (two-tailed Wilcoxon signed-rank test comparing week 3, 10, and 18 to week 0 values) **b** Distances between ASD gut microbiota and donor sample on a per individual basis. Most individuals became more similar to the donor over the study period.
