# Supplementary material for: Microbiota Transfer Therapy alters gut ecosystem and improves gastrointestinal and autism symptoms: an open-label study
Source: Microbiome. 2017 Jan 23;5:10. doi: 10.1186/s40168-016-0225-7 (PMC5264285; doi:10.1186/s40168-016-0225-7)
Supplement: Additional file 4 — Supplementary text. (PDF 123 kb) [file 40168_2016_225_MOESM4_ESM.pdf]

SUPPLEMENTARY INFORMATION

Supplementary Text

Vancomycin/MoviPrep/SHGM. Vancomycin targets the majority of colon bacteria. It has previously been shown to be beneficial in treatment of autism; therefore, it is plausible that vancomycin could be targeting some members of the microbiota that drive the symptoms of autism. Vancomycin is also used to treat *C. difficile* infections. Vancomycin is virtually always used before infusion of fecal microbiota in treatment of recurrent C. difficile infections by fecal microbiota transplantation. It should be noted that this is the only protocol that is currently clearly associated with substantial engraftment of donor microbiota. From standpoint of microbial ecology, vancomycin treatment is needed to “create space” as a conditioning regimen prior to substantial engraftment of a new microbial community.

MoviPrep is a common purgative prior to colonoscopic examinations. The purpose of the bowel lavage here was to ensure clearance of vancomycin from the GI tract, as residual antibiotic could interfere with engraftment of new microbiota. MoviPrep specifically, as opposed to other purgatives, was chosen because of its relatively better tolerability among the general US population undergoing colonoscopic examinations.

SHGM is built upon a body of work with patients being treated for recurrent *C. difficile* infections. Since the preparation of microbiota is standardized, it is the best we can do at this time to build upon this initial work to enable larger trials and attempts at repeating similar regimens by other groups. Our initial dose was comparable to what is used for FMT treatments, and our maintenance dose is comparable to what Thomas Borody used as a

maintenance dose for treating children with ASD (T. Borody, personal communication). Further optimization of dosing is needed in future studies.

Safety/Tolerability/Adverse effects. Children with ASD experienced only temporary adverse effects (primarily mild to moderate hyperactivity and tantrums/aggression) at the beginning of vancomycin treatment, no major changes in blood chemistry or long-term adverse effects were noted. As listed in Additional file 1: Table S3, one participant among the 18 children with ASD (5%) developed an extensive rash, but the rash disappeared when vancomycin was switched from a natural orange flavor to an unflavored form. Within 1-4 days after the start of the vancomycin, 12 children with ASD had a temporary behavioral reaction to the vancomycin either involving hyperactivity (7 out of 12 cases; 39%) or Tantrums/aggression (5 out of 12 cases; 28%). The symptoms lasted 1-3 days in most cases, except for one participant that had symptoms lasting for 3 weeks. After the symptoms disappeared, GI symptoms and behavioral symptoms began improving, which is similar to what Sandler et al. [1] reported in their oral vancomycin therapy for children with autism. Prilosec was generally well-tolerated, but many children had difficulty consuming MoviPrep, due to its taste. Regarding Standardized Human Gut Microbiota (SHGM), rectal administration was remarkably well-tolerated by 6 of 6 recipients. Oral administration of high-dose SHGM was well-tolerated by 12 of 13 recipients, but one participant experienced vomiting and was switched to the rectal route. Oral administration of the low-dose SHGM was well tolerated for 7-8 weeks in all cases.

Participants experienced no major changes in Complete Blood Count (CBC) or blood chemistry panel. Minor changes in blood chemistry were observed as follows. There was a

5% decrease in average levels of potassium (p=0.01) from beginning to end of treatment, but all levels remained in the normal range. After the vancomycin (2nd week of study) there was an 8% increase in platelets (p=0.03), but 4 participants had elevated levels at start, and only 2 participants had elevated levels after vancomycin. A 26% drop in Blood Urea Nitrogen (BUN) (p=0.002) and a 17% increase in Aspartate transaminase (AST) (p=0.01) were observed, although all BUN and AST levels remained in normal range. A 6% increase in albumin/globulin (A/G) ratio (p=0.03) was observed, with 1 slightly elevated. A 24% increase in alanine transaminase (ALT) (p=0.003) was observed, and 1 remained elevated while 2 became slightly elevated over time. However, all these results (platelets, BUN, A/G ratio, AST, ALT) returned to similar to baseline at weeks 5 and 10. Slight changes (1-2%) were observed in levels of mean corpuscular volume (MCV), mean corpuscular hemoglobin (MCH), mean corpuscular hemoglobin concentration (MCHC), and red blood cell distribution width (RDW).

GI symptoms (GSRS and daily stool records). A steady and large degree of improvement in most areas of GSRS evaluation was observed, including abdominal pain, indigestion, diarrhea, and constipation (Additional file 3: Figure S1a). There was little change in reflux since no children had significant reflux at the start of the study. Notably, two seemingly opposite GI symptoms- diarrhea and constipation- responded to the FMT treatment effectively.

The Daily Stool Record (DSR) was collected and averaged it over two weeks in order to assess changes in stool hardness/softness during the study. Overall, a significant decrease was observed in “% days of abnormal stool” that combines % days of hard, soft/liquid, and no

stool, from 62% to 34% (p=0.001) during the 10-week FMT treatment (Additional file 1: Table S2 and Additional file 3: Figure S1b). The improvements remained stable for the following 8 weeks during the observation period. In detail, both “% days of hard stools” (type 1 or 2) and “% days of soft/liquid stools” (type 6 or 7) significantly decreased during the 10-week FMT treatment, but the decrease in “% days of no stool” was not significant (Additional file 1: Table S2).

Autism and Related Symptoms (ABC, VABS-II, and PGI-III). The Aberrant Behavior Checklist (ABC) was employed to assess treatment effects on aberrant behaviors common in children with ASD: irritability, lethargy, stereotypy, hyperactivity, and inappropriate speech. A significant reduction was observed at the end of treatment in all five sub-scales (Additional file 3: Figure S1c).

The Vineland Adaptive Behavior Scale II (VABS-II) is a measure of the functioning level in four different domains: Communication, daily living skills, socialization, and motor skills, based on 11 sub-domains. Among 11 subscales, Fine and Gross Motor skills were excluded, since these two subscales for the Vineland are only calculated up to 6.8 years and most children with ASD started near or over the limit of the scale. The other 9 subscales and their average were compared between baseline and at the end of the study. The FMT treatment resulted in a significant increase in average developmental age, from 5.4 years at baseline to 6.8 years at the end of the study (p<0.001) (Additional file 3: Figure S3). A gain of 1.4 years within 18 weeks of the study is a substantial increase, but they still remained below their chronological age of 10.9 years. Significant improvements were also observed in all 9 subscale areas with the largest gains in interpersonal skills (2.2 years), personal living skills

(1.8 years), and coping skills (1.7 years) (Additional file 3: Figure S3). It is notable that the major impairments in ASD, namely receptive language, expressive language, and interpersonal skills, were among the lowest initial scores, with initial developmental ages of 3.1 years, 4.5 years, and 2.9 years, respectively; all three areas had substantial improvements of 1.3, 1.1, and 2.2 years, respectively.

By the end of the FMT treatment at week 10, the parents rated the change in their children's autism symptoms using the PGI-III, and the largest improvements were in the GI subscore among 17 subscales and "Overall autism/related symptoms" of the PGI-III (Additional file 3: Figure S6). Specifically, the overall scale of PGI-III was rated as Much Better: n= 4 (22%); Better: n= 8 (44%); Slightly Better: n=5 (28%); Little/no change: n=1 (6%). The improvement in the other subscales is shown in Additional file 3: Figure S6.

Stool versus swab microbiome samples. In addition to stool collection over the 18 week period, fecal swab samples were collected nearly every other week. These samples were obtained by swabbing bottoms or used toilet paper with a sterile swab, and thus are easier to collect than stool samples. The general patterns presented for stool data were present in the swab data, but did not always achieve statistical significance in the tests performed for this study (Additional file 3: Figure S7). This may be due to greater variability in the handling of the swab samples, which were shipped to the collection facility at ASU, and thus spent a varied amount of time at ambient temperature, ranging from a few hours to multiple days.

Oral versus rectal administration. No significant difference was observed in efficacy of treatment and changes in gut microbiota after treatment whether FMT was initially

administered rectally or orally (Additional file 3: Figure S8). However, the sample sizes were too small to identify differences, and a larger trial to evaluate the mode of administration should be performed.

Microbiome profiles across even sampling depths and OTU percent identity thresholds. Recent work [2-4] has highlighted important differences in microbiomes that are apparent only by observing differences in presence, absence, and abundance of closely related taxa that may be grouped into single OTUs at the commonly used 97% similarity threshold. To ensure that this study achieved those maximum OTUs, both 100% and 97% OTUs were defined. Analysis focused on the 100% OTUs, but compared microbiome features to those computed based on 97% OTUs to validate the approach. To validate the method, community richness and composition measures were correlated between the 100% and 97% OTUs, such that if analyses were instead performed on 97% OTUs, the results would be reproducible. Faith PD (Pearson r=0.97038, p<0.001, n=569), unweighted UniFrac (Mantel r: 0.92999, p<0.001, n=569), and weighted UniFrac (Mantel r: 0.76651, p<0.001, n=569) were all highly correlated across the two OTU clustering thresholds, suggesting that the same conclusions would be drawn with either approach. The small differences are expected, as there are many more 100% OTUs than 97% OTUs.

Similarly, richness and composition metrics were computed at even sampling (rarefaction) depths of 5,721 and 10,040 on the 100% OTU data. The lower depth allowed us to maximize the number of samples for the analyses, and comparison to the higher depth allows us to confirm that results would be similar if more sequences were retained. Faith PD (Pearson r=0.99738, p<0.001, n=548), unweighted UniFrac (Mantel r: 0.97296, p<0.001), and

weighted UniFrac (Mantel r: 0.99953, p<0.001) were all highly correlated across sampling depths, suggesting that the same conclusions would be drawn based from either sampling depth.

Microbiome results across common diversity metrics. Parallel analyses for multiple diversity metrics were performed to understand the effect of each metric on the findings. With the *Observed OTUs* metric (a count of the number of OTUs observed at least one time in a sample; Additional file 3: Figure S4), the same patterns were observed and presented in Fig. 2a (Spearman rho=0.90, p<0.00001), confirming that both phylogenetic and non-phylogenetic metrics illustrate the same patterns of change in community richness with MTT; i.e., children with ASD initially had lower bacterial richness than controls, but bacterial richness significantly increased after MTT reaching levels closer to those in the neurotypical children. Shannon diversity, a metric which accounts for both the richness and the evenness of samples, also illustrates the same pattern (Spearman rho to Faith PD: 0.83, p<0.00001; Spearman rho to Observed OTUs: 0.96, p<0.00001). These increased bacterial richness remained high 8 weeks after treatment stopped. Increase in bacterial richness was substantial, from an initial median Faith PD value of 47.2 to a final median value of 57.4, an increase of 22%. Similarly, Observed OTUs increased from 1,296 to 1,475, an increase of 12%, and Shannon diversity increased from 7.2 to 7.9, and increase of 9%.

Similarly, pairwise distances were computed between samples using four diversity metrics, a qualitative non-phylogenetic metric (Jaccard distance), and quantitative non-phylogenetic diversity metric (Bray-Curtis distance), a qualitative phylogenetic diversity metric (unweighted UniFrac), and a quantitative phylogenetic diversity metric (weighted

UniFrac) (Additional file 3: Figure S9). Interestingly, while the pattern was the same between the first three metrics (decrease in distance between recipient and donor), significant engraftment with weighted UniFrac was not observed (though less variation across individuals at weeks 10 and 18 than in earlier time points was observed). Quantitative metrics give more weight to higher abundance OTUs than qualitative metrics. Because differences in composition were observed using a quantitative non-phylogenetic metric (Bray-Curtis) but not observed using a quantitative phylogenetic metric (weighted Unifrac), it suggests that when changes occur in high abundance OTUs, those OTUs are generally closely related (thus the change is down-weighted with a phylogenetic diversity metric relative to a non-phylogenetic diversity metric). Overall, 3 of the 4 metrics demonstrate that engraftment occurred, and remained stable at 8 weeks after treatment stopped.

Microbiome results on engraftments using unweighted UniFrac distance. Similarly to data presented in Fig. 3c, when the unweighted UniFrac distance were compared between the recipient gut and their initial donor sample (instead of their most recent/relevant donor sample as in Fig. 3c), the distance also decreased significantly over time (Additional file 3: Figure S10; two-tailed Mann-Whitney U-test $p<0.01$ at three weeks and $p<0.001$ at 10 and 18 weeks) and remained similar to the donor's bacterial community 8 weeks after treatment stopped. In addition, the distance between the recipient gut bacterial community and the major initial donor's was less than the variation of normal interpersonal bacterial community (showed by comparing the neurotypical children variation) at the end of treatment (week 10) as well as 8 weeks after treatment stopped (week 18) (Additional file 3: Figure S10).

References

1. Sandler RH, Finegold SM, Bolte ER, Buchanan CP, Maxwell AP, Vaisanen ML, Nelson MN, Wexler HM: Short-term benefit from oral vancomycin treatment of regressive-onset autism. *Journal of Child Neurology* 2000, 15(7):429-435.
2. Tikhonov M, Leach RW, Wingreen NS: Interpreting 16S metagenomic data without clustering to achieve sub-OTU resolution. *Isme Journal* 2015, 9(1):68-80.
3. Callahan BJ, McMurdie PJ, Rosen MJ, Han AW, Johnson AJ, Holmes SP: DADA2: High-resolution sample inference from Illumina amplicon data. *Nat Methods* 2016.
4. Eren AM, Maignien L, Sul WJ, Murphy LG, Grim SL, Morrison HG, Sogin ML: Oligotyping: differentiating between closely related microbial taxa using 16S rRNA gene data. *Methods in Ecology and Evolution* 2013, 4(12):1111-1119.
